# Translational induction of ATF4 during integrated stress response requires noncanonical initiation factors eIF2D and DENR

**DOI:** 10.1038/s41467-020-18453-1

**Published:** 2020-09-16

**Authors:** Deepika Vasudevan, Sarah D. Neuman, Amy Yang, Lea Lough, Brian Brown, Arash Bashirullah, Timothy Cardozo, Hyung Don Ryoo

**Affiliations:** 1 Department of Cell Biology, New York University Grossman School of Medicine, New York, NY 10016 USA; 2 Department of Pharmaceutical Sciences, University of Wisconsin-Madison, Madison, WI 53705 USA; 3 Department of Biochemistry and Molecular Pharmacology, New York University Grossman School of Medicine, New York, NY 10016 USA

**Keywords:** Stress signalling, Gene regulation, Translation

## Abstract

The Integrated Stress Response (ISR) helps metazoan cells adapt to cellular stress by limiting the availability of initiator methionyl-tRNA for translation. Such limiting conditions paradoxically stimulate the translation of ATF4 mRNA through a regulatory 5′ leader sequence with multiple upstream Open Reading Frames (uORFs), thereby activating stress-responsive gene expression. Here, we report the identification of two critical regulators of such ATF4 induction, the noncanonical initiation factors *eIF2D* and *DENR*. Loss of *eIF2D* and *DENR* in *Drosophila* results in increased vulnerability to amino acid deprivation, susceptibility to retinal degeneration caused by endoplasmic reticulum (ER) stress, and developmental defects similar to *ATF4* mutants. eIF2D requires its RNA-binding motif for regulation of 5′ leader-mediated ATF4 translation. Consistently, eIF2D and DENR deficient human cells show impaired ATF4 protein induction in response to ER stress. Altogether, our findings indicate that *eIF2D* and *DENR* are critical mediators of ATF4 translational induction and stress responses in vivo.

## Abstract (web summary)

Translation of ATF4 mRNA is stimulated during the integrated stress response. Here the authors show that two noncanonical translation initiation factors eIF2D and DENR are required for translational induction of ATF4 mRNA in *Drosophila* and human cells.

## Introduction

The integrated stress response (ISR) in animals and a related-general amino acid control in yeast are adaptive signaling pathways that respond to a variety of stress conditions. Dysregulation of the ISR pathway is associated with a wide variety of diseases ranging from diabetes^1–5^ to neurodegenerative diseases such as Alzheimer’s^6–8^, reflecting the importance of cellular stress adaptation in health. In addition, activation of the ISR aids cancer cell survival and metastasis by enhancing cellular adaptation to extrinsic stresses in the tumor microenvironment^9–11^.

**Fig. 1:**
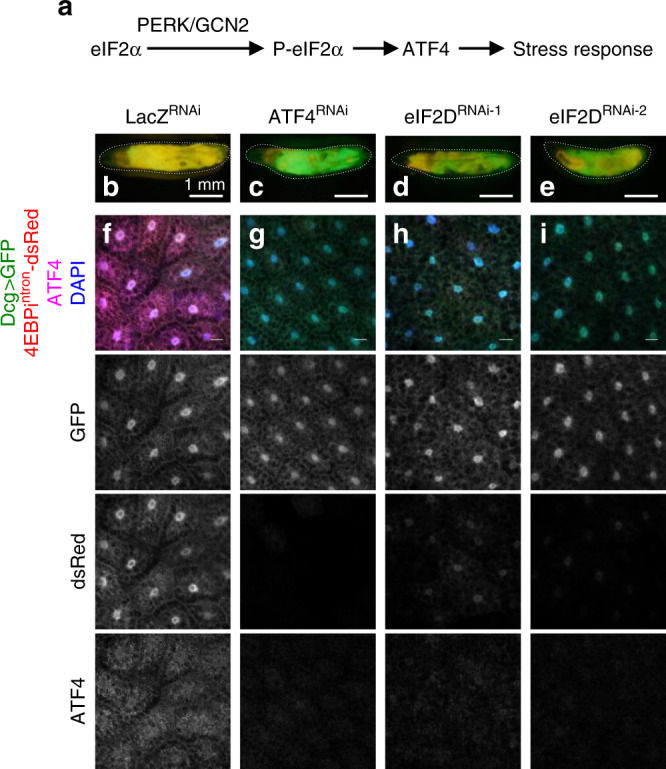
An RNAi screen to identify genes required for ATF4 translation. **a** Line diagram summarizing the ISR pathway in *Drosophila*. **b**–**e** Third instar larvae expressing 4E-BP^intron^-dsRed reporter with indicated RNAi lines and *UAS-GFP* driven by the fat body specific *Dcg-Gal4*. Individual larvae are outlined with dotted lines. Scale bars represent 1 mm. **f**–**i** Fat body tissues dissected from larvae in **b**–**e** expressing GFP (green), dsRed (red), immunolabeled with antiATF4 antibody (magenta) and DAPI (blue). Scale bars represent 25 μm unless otherwise indicated here and in subsequent figures. Data in **b**–**i** are representative images collected from two independent biological experiments with ten animals in each trial.

ISR signaling is initiated by stress-activated kinases that phosphorylate the α-subunit of the eIF2 complex, thereby inhibiting eIF2’s ability to deliver initiator methionyl-tRNA (Met-tRNA_i_^Met^) to ribosomes (Fig. 1a)^12–16^. Such conditions reduce general mRNA translation in cells, but they also stimulate translation of the yeast *GCN4* and the metazoan *ATF4* main open reading frames, which encode transcription factors that mediate a signaling response^12,17,18^. These mRNAs have regulatory 5′ leader sequences containing multiple upstream open reading frames (uORFs), which interfere with main ORF translation in unstressed cells. Upon cellular stress that prompts eIF2α phosphorylation, this 5′ leader sequence now stimulates the main ORF translation (see Supplementary Fig 9 for schematic)^18–22^.

Thus far, eIF2 has been the only Met-tRNA_i_^Met^ delivering factor implicated in initiation of ATF4 translation, and how ATF4 ORF translation is induced under limiting eIF2 conditions has remained poorly understood. Here we report the identification of a noncanonical methionyl-tRNA-delivering initiation factor, *eIF2D*, as a gene required for the translation of ATF4 in vivo. *eIF2D* is partially redundant with its homolog *DENR* in both *Drosophila* and in human cells. eIF2D requires its RNA-binding motif to regulate the ATF4 5′ leader. Loss of *eIF2D* and *DENR* in *Drosophila* phenocopies the pupal developmental defects observed in mutants of *cryptocephal (crc)*, the *Drosophila ATF4*. In addition, *eIF2D* and *DENR* mutants are more vulnerable to amino acid deprivation and susceptible to age-dependent retinal degeneration in a *Drosophila* model of Retinitis Pigmentosa. Altogether, our findings indicate that noncanonical initiation factors are required for ISR signaling in vivo and provide insights into how ATF4 is translationally induced in stressed cells with attenuated general translation.

## Results

### eIF2D is a regulator of ATF4 mRNA translation

*4E-BP* is an established transcriptional target of ATF4 in both *Drosophila* and mammals^10,23–26^. Like mammals, *Drosophila* cells detect stress through conserved eIF2α kinases that activate ATF4-mediated ISR signaling^27–29^. We previously generated a transgenic *Drosophila* line in which the ATF4 responsive element in *4E-BP* (*4E-BP*^*intron*^) drives the expression of *dsRed*. This line reports physiological ATF4 signaling in the late third instar larval fat body, an adipose-like tissue present underneath the cuticle of the larval torso^24^ (Fig. 1b, f). *4E-BP*^*intron*^*-dsRed* is also expressed in the salivary glands located at the anterior end of each larva, but this signal is not affected by loss of *ATF4*^24^. Thus, we focused on the dsRed fluorescence in the fat body to assess ATF4 signaling activity.

To identify genes required for ATF4 signaling, we drove expression of *UAS-RNAi* lines targeting genes annotated as translation initiation factors using the fat body-specific *Dcg-Gal4* driver (Supplementary Table 1). We also co-expressed *UAS-GFP* together as a control. We selected for RNAi lines that suppressed *4E-BP*^*intron*^*-dsRed* expression (reporting ATF4 activity) but had no effect on control *GFP* expression (reporting general cellular translation). As we reported previously^24^, the RNAi line targeting *ATF4* caused a loss of dsRed but no change in GFP (Fig. 1c, g). Of the 50 RNAi lines targeting 28 genes, one of the two that targeted *Pdcd4* (Programmed cell death 4, FlyBase ID: FBgn0030520) and two lines targeting *eIF2D* (eukaryotic Initiation Factor 2D, FlyBase ID: FBgn0041588) reduced *4E-BP*^*intron*^*-dsRed* expression in the fat body (Fig. 1d, e and Supplementary Table 1). The two independent *eIF2D* RNAi lines targeted different regions of the gene, and therefore, we considered *eIF2D* a high confidence hit. The results from the screen were confirmed in dissected fat body tissues, which had decreased 4E-BP^intron^-dsRed signal upon knockdown of *eIF2D* without affecting control *GFP* expression (Fig. 1h, i). Additional immunolabeling with anti-ATF4 showed that *eIF2D* knockdown reduced ATF4 protein levels in the fat body (Fig. 1h, i). These experiments suggest that *eIF2D* is required for ATF4 expression in the fat body, but not for general mRNA translation.

**Fig. 2:**
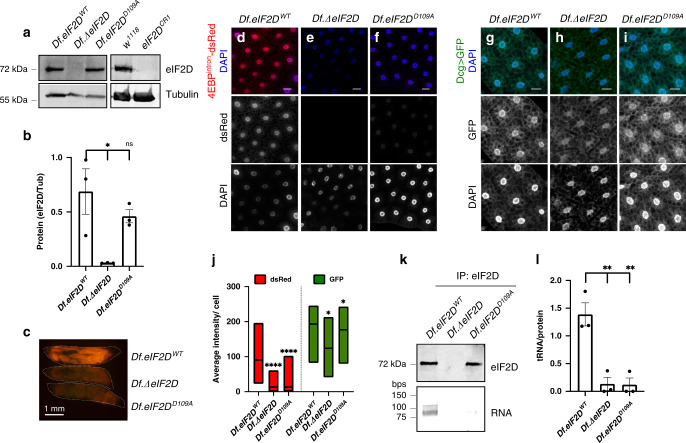
*eIF2D* is required for ATF4 expression and ISR signaling. **a** Western blot analysis of larval extracts from various allelic combinations of *eIF2D* mutants. *w*^*1118*^ is the isogenic wild type control for *eIF2D*^*CR1*^. **b** Quantitation of data from western blots in **a** representing the mean of three independent biological replicates with error bars representing standard error. **c** Expression of 4E-BP^intron^-dsRed in control larvae (*Df.eIF2D*^*WT*^), *eIF2D* deletion mutant (*Df.ΔeIF2D*) and an eIF2D mutant where the tRNA-binding interface is disrupted (*Df.eIF2D*^*D109A*^). Individual larvae are shown in dotted outlines. **d**–**f** Fat body tissues dissected from larvae in **c** showing 4E-BP^intron^-dsRed (red), and counterstained with DAPI (blue). **g**–**i** Fat body tissues from larvae of indicated genotypes expressing GFP (green) driven by the fat body specific driver, *Dcg-GAL4*, and counterstained with DAPI (blue). **j** Quantitation of fluorescent protein intensity from individual cells in **d**–**f** and **g**–**i**. Midline represents the mean value, with the top and bottom of the box representing the maximum and minimum values. Asterisks above boxes represent statistical significances between mutant and control values. *n* = 278, 341, 231, 213, 335, and 395, respectively for each box from left to right. **k** Analysis of eluates from immunoprecipitation of eIF2D. Top panel shows western blot analysis of eluates and bottom panel shows TBE-UREA gel analysis of bound RNA. **l**. Quantification of data from **k** representing the mean of four biological replicates, with RNA levels for each sample normalized to respective protein levels. Data in **c**–**i** are representative images collected from two independent biological experiments with ten animals in each trial. Statistical significance in **b**, **j**, and **l** were calculated using the two-tailed t-test with ****p < 0.00001, **p < 0.001, *p < 0.01 and n.s. not significant. Please see Source Data Files for the raw data in **a**, **b**, **j**, **k**, **l**.

**Fig. 3:**
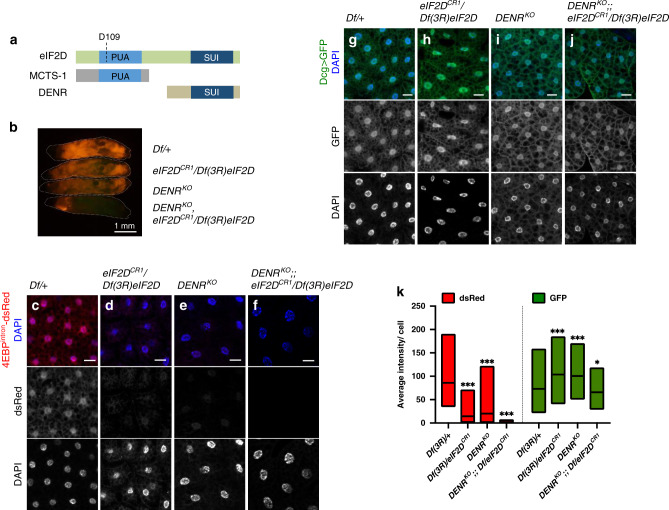
*eIF2D* shares its function with *DENR*. **a** A schematic diagram showing the domain architecture of eIF2D, DENR, and MCTS-1. **b** Expression of 4E-BP^intron^-dsRed in *eIF2D* and *DENR* single mutants (*DENR*^*KO*^) compared with that of *DENR; eIF2D* double mutants. Individual larvae are indicated with dotted outlines and their genotypes are indicated on the right. **c**–**f** Fat body tissues dissected from larvae in **b** showing 4E-BP^intron^-dsRed (red) and DAPI (blue). **g**–**j** Fat body tissues from larvae of indicated genotypes expressing GFP (green) driven by the fat body specific driver, *Dcg-GAL4*, and counterstained with DAPI. **k** Quantitation of fluorescent protein intensity from individual cells in **c**–**j**. Midline represents the mean value, with the top and bottom of the box representing the maximum and minimum values. Asterisks above boxes represent statistical significances between mutant and control values calculated with the a two-tailed *t*-test with ***p < 0.0001 and *p < 0.01. *n* = 384, 370, 426, 362, 103, 158, 145, and 138, respectively for each box from left to right. Data in **b**–**j** are representative images collected from three independent biological experiments with seven animals in each trial. Please see Source Data Files for the raw data used to generate graph in **k**.

To validate our data from RNAi experiments, we generated two independent mutant alleles of *eIF2D*. We deleted a large genomic locus that spans *eIF2D* to generate a “Deficiency” line, *Df(3R)eIF2D* (Supplementary Fig. 1a). This deficiency was complemented with a genomic rescue transgene that deletes only the *eIF2D* sequence to generate the *eIF2D* mutant, *Df.ΔeIF2D* (Supplementary Fig. 1a; see “Methods” section). An equivalent line with a wild-type *eIF2D* rescue transgene, *Df.eIF2D*^*WT*^, was also generated for comparison. A second allele was made using CRISPR–Cas9 gene editing (*eIF2D*^*CR1*^, Supplementary Fig. 1b). Both alleles abolished *eIF2D* expression as detected through western blots (Fig. 2a, b). Consistent with the RNAi data, *eIF2D* mutants showed reduced ATF4 pathway activity as assessed by an 85% decrease in 4E-BP^intron^-dsRed in the fat body (Fig. 2c–e, j and Supplementary Fig. 1c–e). In contrast, loss of *eIF2D* had only a very minor effect on the expression of a control *GFP* transgene (Fig. 2g, h, j). We ascribe this minor change to variations in genetic background because it was not reproducible in the *Df(3R)eIF2D/eIF2D*^*CR1*^ transheterozygote (see Fig. 3k). Thus, we conclude that the effect of *eIF2D* loss is specific for the ATF4 signaling reporter, 4E-BP^intron^-dsRed.

To further confirm these results, we attempted to rescue the loss of *4E-BP*^*intron*^*-dsRed* expression in *eIF2D* mutants by ectopically expressing a transgene containing the *Drosophila* ATF4 coding sequence without the 5′ leader (*crc*^*leaderless*^) to ensure ATF4 expression independent of upstream regulators. Fat body-specific expression of *crc*^*leaderless*^, but not a control transgene (*lacZ*), in *eIF2D* mutants restored the expression of *4E-BP*^*intron*^*-dsRed* (Supplementary Fig. 2). Notably, overexpression of *ATF4* with the *crc*^*leaderless*^ transgene resulted in fragile fat body tissues in both control and *eIF2D* mutant animals, and led to an increase in *4E-BP*^*intron*^*-dsRed* expression even in control animals (Supplementary Fig. 2a, b, d, f).

### eIF2D requires its RNA-binding motif for ATF4 regulation

eIF2 has been the only known Met-tRNAi^Met^-delivering translational initiation factor implicated in ATF4 mRNA translation. At the same time, it has been established in yeast and mammals that translation of the GCN4 and ATF4 main ORF requires eIF2 inhibition^17,30^. Under these conditions, it is assumed that residual eIF2 activity mediates translation initiation of the ATF4 main ORF. Identification of *eIF2D* as an ATF4 regulator was intriguing, as previous studies have shown that eIF2D can deliver Met-tRNA_i_^Met^ to P-sites of ribosomes on AUG start codons in vitro^31–33^. eIF2D does not share sequence homology with eIF2; it contains a PUA domain capable of interacting with Met-tRNA_i_^Met^, and a SUI1 domain that can recognize start codons^31,32^. Based on the available crystallographic structures of RNA-bound PUA domains and the cryoEM structure of eIF2D bound to uncharged tRNA (PDB:5oa3), we generated a third eIF2D mutant line with an amino acid residue substitution at the PUA domain:RNA binding interface (*Df.eIF2D*^*D109A*^, Fig. 2a and Supplementary Fig. 3a, also see Fig. 3a). The D109A mutant protein was expressed at similar levels to wildtype *eIF2D* as assessed by western blots from larval tissues (Fig. 2b).

The human equivalent of D109 is D111, which is close to the first cytosine (C) of the universally-conserved CCA tail of the tRNA, likely to help stabilize the bound tRNA (Supplementary Fig. 3b)^33,34^. The D109A mutation was designed to specifically diminish tRNA binding without destabilizing the whole PUA fold. To test this, we immunoprecipitated eIF2D from wild-type control larvae (*Df.eIF2D*^*WT*^) and found nucleic acid species roughly the size of tRNA that co-purified with the eIF2D protein (Fig. 2k). No such species were detected in the eIF2D deletion mutants (*Df.ΔeIF2D)* (Fig. 2k, l). The co-purifiying nucleic acid species was DNase-insensitive but RNase-sensitive (Supplementary Fig. 3c), indicating that eIF2D binds an RNA molecule. Consistent with our predictive modeling, the D109A mutant protein failed to copurify with the RNA species that was associated with the wild-type protein (Fig. 2k, l). We also found evidence that the *eIF2D*^*D109A*^ mutation disrupts ATF4 signaling. In comparison to the control *Df.eIF2D*^*WT*^ genotype, the 4E-BP^intron^-dsRed reporter was significantly diminished in the *Df.eIF2D*^*D109A*^ mutants (Fig. 2c, f, j) without significantly affecting the expression of a control *GFP* transgene (Fig. 2i, j). We note that the D109A allele had a slightly reduced effect on the ATF4 pathway reporter in comparison to the null allele (Fig. 2j). We speculate that this could be due to trace amounts of RNA binding activity in the D109A mutant that we could not detect in our experiments.

### *eIF2D* shares its function with *DENR*

Although *eIF2D* mutants show a substantial decrease in 4E-BP^intron^-dsRed, we did not see a complete loss in the reporter signal (Fig. 2c, j and Supplementary Fig. 1c), suggesting that other factors act redundantly with eIF2D. For this reason, we turned our attention to Density-regulated reinitiation and release factor (*DENR*) and Multiple copies in T-cell lymphomas-1 (*MCTS-1*), two other proteins that contain SUI and PUA domains respectively (Fig. 3a). *DENR* and *MCTS-1* have been implicated in regulating translation reinitiation of uORF containing transcripts that regulate growth and development in nonstressed cells^35^. In vitro biochemical assays show that eIF2D has the same function as the DENR–MCTS1 complex, that as a heterodimer reconstitutes the domain architecture of eIF2D^31,34,36,37^. While there are no available *MCTS-1* loss-of-function mutants, a null *DENR*^*KO*^ strain was reported previously^35^. In this mutant background, we saw reduction of *4E-BP*^*intron*^*-dsRed* expression like that seen in *eIF2D* mutants (Fig. 3b–e). Strikingly, double mutants for *eIF2D* and *DENR* showed a complete loss of 4E-BP^intron^-dsRed in the fat body (Fig. 3b, c, f), with only ATF4-independent dsRed signals remaining in the salivary glands near the anterior end of the larvae. Like our analyses with *eIF2D* mutants, we attempted to rescue this loss of 4E-BP^intron^-dsRed by ectopic expression of the *crc*^*leaderless*^ transgene in the fat body. When raised at either 25 or 20 °C, we could not recover *eIF2D DENR* double mutants overexpressing ATF4. However, we observed a complete rescue of 4E-BP^intron^-dsRed in *DENR*^*KO*^ *eIF2D*^*CR1*^/+ animals raised at 20 °C (Supplementary Fig. 4). These results indicate that *eIF2D* and *DENR* both contribute to ATF4 signaling.

**Fig. 4:**
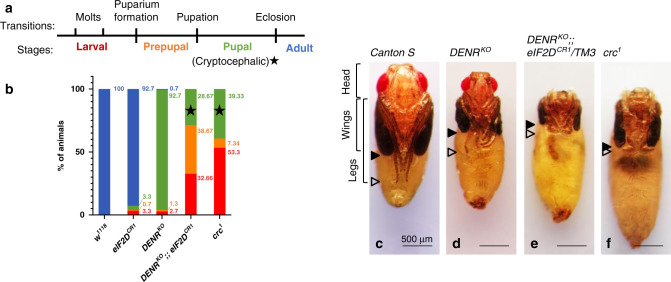
*DENR eIF2D* double mutants resemble *ATF4* mutants. **a** Schematic of developmental transitions during the *Drosophila* life cycle. **b** Lethal phase analysis for control, *eIF2D*, *DENR*, *DENR eIF2D* double, and *crc* animals. Developmental stages are color-coded as shown in the schematic in **a**. Star symbol indicates animals that arrest during metamorphosis as cryptocephalic pupae. *n* = 150 for each genotype. **c**–**f** Analysis of pupal morphology in dissected control (*Canton S)*, *DENR*, *DENR eIF2D/TM3*, and *crc* mutant animals. Solid arrowheads indicate the degree of wing extension and outlined arrowheads indicate the extent of leg extension. An extended analysis of lethal phase and animal morphology can be found in Supplementary Information (Extended analysis of lethal phase and animal morphology). Data in **c**–**f** are representative images collected from two independent biological experiments with ten animals in each trial. Scale bars are 500 μm.

We compared the phenotypic consequences of *DENR* and *eIF2D* loss-of-function with that of the *Drosophila ATF4* ortholog, *cryptocephal* (*crc*). *crc* is so named because the mutant animals exhibit a cryptocephalic, or hidden head phenotype, characterized by complete absence of a head and failure to extend the wings and legs^38^ during pupation (*c.f*. Fig. 4c vs. f), the developmental transition between prepupa and pupa (Fig. 4a). The cryptocephalic phenotype is extremely rare, with only five other loss-of-function *Drosophila* mutant alleles reported to exhibit this defect at a low penetrance (*Eip74EF*^*Pneo24*^, *br*^*rbp-5*^, *ftz-f1*^*ex17*^, *Kr-h1*^1^, and *Sox14*^*L1*^; Flybase)^39–43^. These five genes are conserved regulators of ecdysone signaling during insect metamorphosis. Importantly, *DENR eIF2D* double mutant animals, like *crc* mutant animals, exhibit a fully penetrant cryptocephalic phenotype (Fig. 4b). Loss of even one allele of *eIF2D* in *DENR* mutants resulted in a cryptocephalic phenotype (Fig. 4e), underscoring the contribution of both genes to ATF4 expression during development. *crc* and *DENR eIF2D* double mutants also exhibit other striking similarities, including lethality during larval stages (Fig. 4b) and defects during puparium formation (Supplementary Fig. 5a, d, e). In contrast, neither *DENR* nor *eIF2D* single mutants recapitulate *crc* mutant phenotypes. *eIF2D* single mutant animals are viable and do not exhibit any obvious developmental defects (Fig. 4b and Supplementary Fig. 5b), while *DENR* mutant animals primarily arrest during pupal stages of development^35^ (Fig. 4b) with minor defects in pupation (Fig. 4d and Supplementary Fig. 5c). The difference in severity between *eIF2D* and *DENR* mutants suggest that these factors may act in a tissue-specific manner and/or have other essential targets, a few of which are reported for DENR^35,44,45^. Taken together, our results demonstrate that the *DENR eIF2D* double mutant phenotype bears marked resemblance to the *crc* mutant phenotype, suggesting that *DENR* and *eIF2D* function redundantly to regulate ATF4 signaling during development.

### Loss of *eIF2D* and *DENR* results in impaired stress responses

**Fig. 5:**
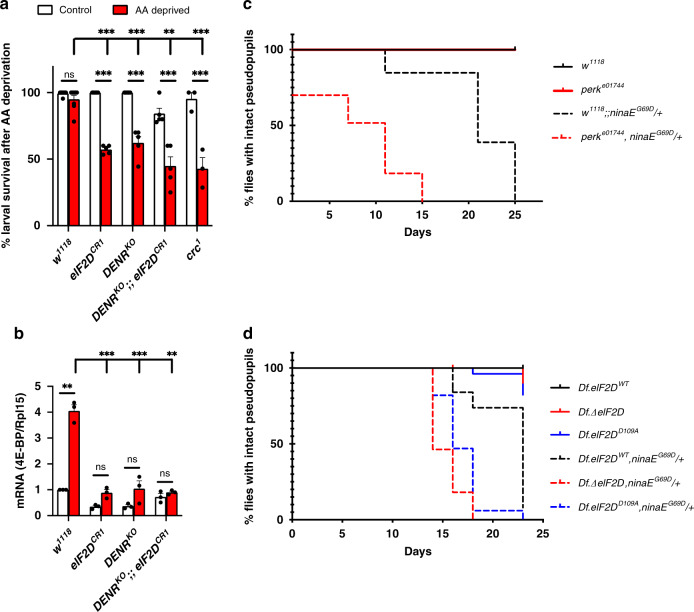
*eIF2D* and *DENR* modulate stress response phenotypes. **a** Survival rate of second instar larvae of the indicated genotypes when fed with normal food (white bars) or amino acid deprived food (4% sucrose, red bars) for 8 h. Data represent the mean from five independent experiments with 20 animals in each trial, and error bars represent standard error. **b** qPCR analysis of *4E-BP* mRNA levels in larvae from **a**. Data are the mean from three independent experiments, with error bars representing standard error. In both **a, b**, *p* values were calculated using the two-tailed *t*-test with **p < 0.001, ***p < 0.0001 and n.s. not significant. **c** Photoreceptor degeneration in the *ninaE*^*G69D*^*/+* adRP model in control and *perk* mutants as assessed by *Rh1-GFP* fluorescence that allows for visualization of photoreceptors in adult pseudopupils. Note that the lines for *w*^*1118*^ (solid black) and *perk*^*e01744*^ (solid red) overlap. The difference in the course of retinal degeneration between the following pairs is statistically significant as assessed by the Log-rank (Mantel-Cox) test (*p* < 0.0001): *w*^*1118*^ and *w*^*1118*^*;;ninaE*^*G69D*^*/+*, *perk*^*e01744*^ and *perk*^*e01744*^*,ninaE*^*G69D*^*/+*, *w*^*1118*^*;;ninaE*^*G69D*^*/+* and *perk*^*e01744*^*,ninaE*^*G69D*^*/+*. (*n* = 100). **d** Photoreceptor degeneration with *ninaE*^*G69D*^*/+* monitored in various *eIF2D* mutant backgrounds. Note that the curves for the control *Df.eIF2D*^*WT*^ (solid black), *Df.ΔeIF2D* (solid red) and *Df.eIF2D*^*D109A*^ (solid blue) overlap for the early time points. The difference in the course of retinal degeneration between the following pairs is statistically significant as assessed by Log-rank (Mantel-Cox) test (*p* < 0.0001): *Df.eIF2D*^*WT*^ and *Df.eIF2D*^*WT*^*,ninaE*^*G69D*^*/+, Df.ΔeIF2D* and *Df.ΔeIF2D,ninaE*^*G69D*^*/+*, *Df.ΔeIF2D* and *Df.ΔeIF2D,ninaE*^*G69D*^*/+*, *Df.eIF2D*^*WT*^*,ninaE*^*G69D*^*/+* and *Df.ΔeIF2D,,ninaE*^*G69D*^*/+*, *Df.eIF2D*^*WT*^*,ninaE*^*G69D*^*/+ and Df.eIF2D*^*D109A*^*,ninaE*^*G69D*^*/+*. (*n* = 100). Please see Source Data Files for the raw data in **a**–**d**.

To determine the physiological role of *eIF2D* in stress responses, we subjected wild type and *eIF2D* mutant flies to amino acid deprivation, a condition that activates the eIF2α kinase GCN2, culminating in the transcriptional induction of *4E-BP* by ATF4^24^. Under normal feeding conditions, most *eIF2D*^*CR1*^and *DENR*^*KO*^ single or double mutant second instar larvae survived at high rates in the 8-h window of inspection. However, the survival rate decreased significantly in these mutants when grown on amino acid-deprived food for 8 h, comparable to that observed in the *ATF4* mutant, *crc*^*1*^ (Fig. 5a). This reduced survival was consistent with the impaired transcriptional induction of *4E-BP* (Fig. 5b). Notably, deletion of both *eIF2D* and *DENR* resulted in a ~50% decrease in ATF4 mRNA (Supplementary Fig. 6a), suggesting destabilization of the ATF4 transcript in the absence of these translation factors. However, the reduction in ATF4 mRNAs is modest, compared to the ~95% reduction in the ATF4 reporter (4E-BP^intron^-dsRed) expression (Fig. 3k). We further confirmed that reducing ATF4 gene dosage in half by using *crc*^*1*^ heterozygous mutants did not affect *4E-BP*^*intron*^*-dsRed* expression in the fat body (Supplementary Fig. 6b–d).

To examine the role of *eIF2D* in a pathological context, we employed *a Drosophila* model for autosomal dominant retinitis pigmentosa (adRP), which utilizes a dominant point mutation in the endogenous Rh-1 encoding gene, *ninaE* (*ninaE*^*G69D*^*)*^46,47^. These mutations are analogous to the Rhodopsin mutants that underlie human adRP^48,49^. The misfolding-prone Rh-1^G69D^ protein encoded by the *Drosophila ninaE*^G69D^ allele imposes endoplasmic reticulum (ER) stress and triggers age-dependent retinal degeneration similar to the human disease^50,51^. Misexpression of Rh-1^G69D^ in larval imaginal disks results in induction of ATF4 protein^52^ and its downstream target *4E-BP*^24^. However, the role of ATF4 signaling in age-related retinal degeneration of *ninaE*^*G69D*^ mutant adults has not been established. To do so, we examined PERK, the eIF2α kinase required for ATF4 induction in response to ER stress^17^. As mentioned above, *crc*^*1*^ mutants arrest during development, but loss-of-function *perk* mutants (*perk*^*e01744*^*)*^29^ are viable and therefore amenable for age-related retinal degeneration analysis. Using the *Rh1-GFP* reporter that marks intact retinal photoreceptors (see “Methods” section), we observed that *perk* homozygous mutants in a *ninaE* wild type background did not show any signs of age-related retinal degeneration (Fig. 5c). As reported previously^50,53^, flies bearing the heterozygous *ninaE*^*G69D*^ mutation exhibit photoreceptor degeneration between from 12 to 25 days after eclosion. The course of retinal degeneration in the *ninaE*^*G69D*^ heterozygous flies was dramatically accelerated in *perk*^*e01744*^ animals, with approximately 30% of flies exhibiting retinal degeneration at eclosion, and all flies showing retinal degeneration by day 15 (Fig. 5c). These results indicate that ISR signaling plays a protective role during retinal degeneration in response to ER stress.

We next examined if *eIF2D* affects retinal degeneration in the *ninaE*^*G69D*^ model. Compared to control *Df.eIF2D*^*WT*^ flies bearing the heterozygous *ninaE*^*G69D*^ mutation, *Df.ΔeIF2D* mutant flies in an otherwise equivalent genetic background showed an early onset of degeneration at 14 days, with the data being statistically significant at *p* < 0.0001 as measured by the log rank analysis (Fig. 5d). Consistent with observations using *4E-BP-dsRed* (Fig. 2c–f), the *Df.eIF2D*^*D109A*^ RNA-binding mutant allele showed an intermediate phenotype in the *ninaE*^*G69D*^ heterozygous background, with the majority of the flies undergoing degeneration at 16 days (Fig. 5d). The accelerated retinal degeneration phenotype of *eIF2D* mutants is similar to that observed in *perk* mutants, albeit less severe, likely due to the presence of DENR–MCTS1. The adult retinal degeneration phenotype could not be tested in *DENR*^*KO*^ animals which are pupal lethal, as described above. None of the control or mutant alleles showed retinal degeneration in the absence of the *ninaE*^*G69D*^ allele (Fig. 5d). These data indicate that *eIF2D* plays a protective role in ER stress-induced photoreceptor degeneration.

### eIF2D and DENR control gene expression through the ATF4 5′ leader

**Fig. 6:**
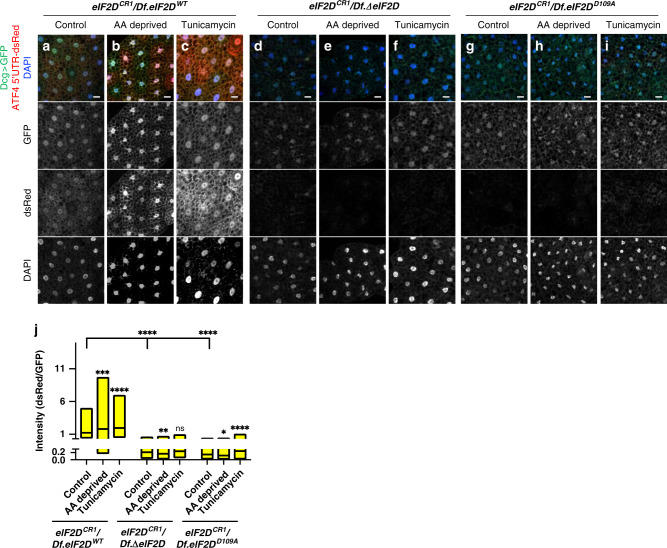
*eIF2D* and *DENR* regulate the translation of the ATF4 ORF. **a**–**i** Expression of the ATF4 5′UTR-dsRed reporter in fat bodies of control (*Df.eIF2D*^*WT*^), *eIF2D* transheterozygous null (*Df.ΔeIF2D*) and point (*Df.eIF2D*^*D109A*^) mutants. The reporter utilizes a dsRed reporter bearing a nuclear localization sequence. Control GFP expression is driven by *Dcg-GAL4*. Larvae in **b**, **e**, **h** were subjected to 4 h of amino acid deprivation and those in **c**, **f**, **i** were fed Tunicamycin for 4 h. Data are representative images collected from two independent biological experiments with ten animals in each trial. **j** Quantification of the ATF4 5′UTR-dsRed reporter intensities normalized to corresponding GFP intensities from the same cells. Midline represents the mean value, with the top and bottom of the box representing the maximum and minimum values. Asterisks above the boxes represent statistical significances between mutant and control values calculated with the a two-tailed *t*-test with ***p < 0.0001 and *p < 0.01. *n* = 158, 157, 112, 104, 139, 131, 120, 148, and 137, respectively for each box from left to right. Please see Source Data Files for raw data.

Since *eIF2D* was originally characterized as a putative translation initiation factor^31,32^, we sought to test if it regulates the translation of *ATF4* mRNA via its 5′ leader. Using a reporter, where the coding sequence of *ATF4* is replaced with nuclear localization sequence-tagged *dsRed* and expressed via a tubulin promoter (*ATF4 5*′*UTR-dsRed*)^54^, we found that *Df.ΔeIF2D* and *Df.eIF2D*^*D109A*^ mutant larvae have reduced nuclear dsRed signals in the fat body without affecting control GFP expression (Fig. 6a, d, g, j), indicating that the effect of *eIF2D* loss is specific to our reporter with the ATF4 5′ leader. Loss of *DENR* showed a similar reduction in the expression of the *ATF4 5*′*UTR-dsRed* reporter (Supplementary Fig. 7), indicating that eIF2D and DENR likely regulate ATF4 mRNA translation via the ATF4 5′ leader. We also tested the effect of eIF2D on the induction of ATF4 via its 5′UTR in response to stress imposed by amino acid deprivation or Tunicamycin^54^. We found that both *Df.ΔeIF2D* and *Df.eIF2D*^*D109A*^ mutant larvae showed little to no induction of ATF4 5′UTR-dsRed in response to either amino acid deprivation (Fig. 6b, e, h, j) or Tunicamycin feeding (Fig. 6c, f, i, j), while control larvae (*Df*.*eIF2D*^*WT*^) showed an expected increase in *dsRed* expression in both conditions (Fig. 6j). There was little to no effect on expression of a control *GFP* transgene in any of the conditions or genotypes tested (Fig. 6a–i).

### Human cells require eIF2D and DENR for stress-induced ATF4 induction

**Fig. 7:**
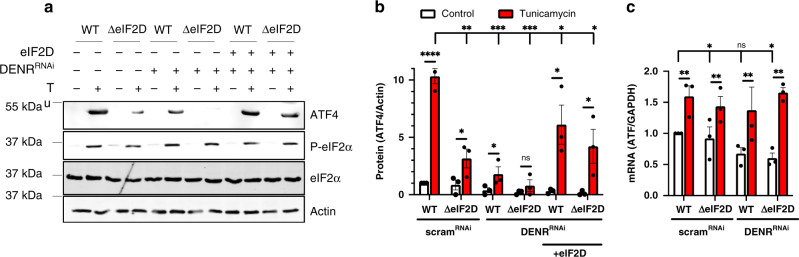
Regulation of ATF4 by *eIF2D* and *DENR* is conserved in human cells. **a** Western blots of total cell lysates from control human HAP1 cells (WT) or equivalent cells with CRISPR-Cas9 mediated deletion of the eIF2D locus (ΔeIF2D). Cells were transfected with scrambled RNAi or DENR RNAi, and treated with DMSO or Tunicamycin (Tu, 10 μg/ml). pcDNA-heIF2D was used to re-introduce human eIF2D into cells. The blots were probed with antibodies recognizing ATF4 (panel 1), phospho-eIF2α (panel 2), total eIF2α (panel 3), and actin (panel 4). **b** Quantitation of ATF4 protein levels in **a** as normalized to the loading control (actin). Data are the mean of 3 independent experiments. **c** qPCR analysis of ATF4 mRNA in HAP1 cells from **a** normalized to GAPDH. Data are the mean of three independent experiments. Error bars represent standard error, *p* values were calculated using the two-tailed *t*-test with *p < 0.01, **p < 0.001, ****p < 0.00001 and n.s. not significant. Please see Source Data Files for raw data in **a**–**c**.

To determine if the role of eIF2D in ATF4 translation is conserved in humans, we assessed ATF4 expression in HAP1 cells (a near haploid human cell line), where *eIF2D* was deleted using CRISPR-Cas9 editing (ΔeIF2D). As expected, treatment with the ER stress-causing chemical Tunicamycin induced ATF4 protein synthesis in wild type (WT*)* control cells, while ΔeIF2D HAP1 cells showed a partial reduction in ATF4 induction under otherwise equivalent conditions (Fig. 7a, b and Supplementary Fig. 8a, b). Knocking down DENR in WT HAP1 cells also resulted in a partial decrease in ATF4 induction in response to Tunicamycin treatment (Fig. 7a, b). Consistent with the *Drosophila* system, knocking down DENR in ΔeIF2D HAP1 cells (Supplementary Fig. 8c, d) led to a substantial loss of ATF4 induction, more so than individually depleting eIF2D or DENR (Fig. 7a, b). While these conditions blocked ATF4 synthesis, they did not reduce eIF2α phosphorylation (Fig. 7a and Supplementary Fig. 8a, e), ruling out the possibility that eIF2D and DENR indirectly affect ATF4 through upstream factors. The loss of ATF4 induction in cells lacking eIF2D and DENR was rescued by transgenic expression of eIF2D, ruling out potential off-target effects of CRISPR-Cas9 or DENR siRNA (Fig. 7a, b). As reported previously, induction of stress led to a moderate increase in ATF4 mRNA levels^55^, which is also seen in ΔeIF2D HAP1 cells and WT cells treated with DENR siRNA (Fig. 7c). While depletion of both DENR and eIF2D saw a ~40% decrease in ATF4 mRNA levels in unstressed cells, ATF4 mRNA amount in Tunicamycin-treated cells were not significantly different (Fig. 7c), consistent with a requirement for eIF2D and DENR in ATF4 mRNA translation during stress.

## Discussion

Here, we report *eIF2D* and *DENR* as previously unrecognized regulators of ATF4 translation in *Drosophila* and human cells. Though several studies have examined regulatory targets of DENR individually^35,44^, none have uncovered the link between these translational initiation factors and ATF4-mediated ISR signaling. Our data show that in the absence of both eIF2D and DENR, ATF4 fails to be induced in *Drosophila* and human HAP1 cells. Furthermore, we demonstrate that the loss of *eIF2D* and *DENR* disrupts a number of pathophysiological processes in *Drosophila* that also require ATF4 signaling.

Previously reported biochemical functions of eIF2D and DENR-MCTS-1 as noncanonical Met-tRNA_i_^Met^ delivering translation initiation factors^32,56^ provide mechanistic insights regarding the translational regulation of ATF4. For mRNAs with single ORFs in their 5′ leaders, the AUG codon procures the first methionine through ribosomes associated with the eIF2-GTP-Met-tRNA_i_^Met^ ternary complex^21^. However, during translation of mRNAs with multiple uORFs, such as those seen in the ATF4 5′ leader (schematic in Supplementary Fig. 9), the scanning ribosome consumes the Met-tRNA_i_^Met^ upon encountering the uORFs. Therefore, translation initiation at the ATF4 main ORF requires the scanning ribosomes to acquire another Met-tRNA_i_^Met^. We posit that eIF2D and DENR (together with MCTS-1) could act as Met-tRNA_i_^Met^ delivery factors for the ATF4 ORF (model in Supplementary Fig. 9). This is supported by previous in vitro experiments showing that eIF2D can act as a noncanonical Met-tRNA_i_^Met^ carrier^31,56^, and by our data showing that the RNA binding interface within the PUA domain of eIF2D is required for regulation of ATF4 via the 5′ leader. This model of eIF2D and DENR–MCTS1 acting as noncanonical Met-tRNA_i_^Met^ carriers would predict a decreased occupancy of ribosomes at the ATF4 main ORF in the absence of eIF2D and DENR-MCTS1. An accompanying manuscript by Teleman and colleagues^57^ also independently reports that loss of DENR negatively affects ATF4 translation due to decreased ribosome occupancy at the ATF4 ORF, validating our hypothesis. These data support our model that eIF2D and DENR affect translation of ATF4, and warrant future studies to further understand the molecular mechanism of this model.

eIF2D and DENR-MCTS-1 have been demonstrated to also participate in ribosome recycling after translation termination^31,56,58^. If this were the primary function of eIF2D and DENR-MCTS-1 in translation of the ATF4 mRNA, decreased ribosome recycling in *eIF2D DENR* double mutants would be predicted to have the effect of enhancing ATF4 main ORF translation. In *Saccharomyces cerevisae*, the homologs of *eIF2D*, *DENR,* and *MCTS-1* (Tma64 Tma22, and Tma20, respectively) have more prominent roles in ribosome recycling as evidenced by increased translation of downstream of ORFs and uORFs^58^. The translation of GCN4, the *S. cerevisiae* equivalent of ATF4, also increases upon the loss of these recycling factors^59^. Together, these findings suggest that the in vivo roles of eIF2D, DENR, and MCTS have diverged during evolution, with metazoan factors playing more prominent roles in translational initiation of ATF4 mRNA.

In conclusion, we report that unconventional Met-tRNA_i_^Met^ delivery factors are required for ATF4 induction during ISR signaling in *Drosophila* and humans. This discovery provides insights into how ATF4 translation occurs efficiently in stressed cells, where the canonical Met-tRNA_i_^Met^ delivery factor, eIF2, is inhibited by eIF2α kinases. Additionally, since abnormal ISR signaling is associated with a wide range of metabolic and degenerative diseases in humans, we anticipate these findings to have clinical impact.

## Methods

### Drosophila genetics

All *Drosophila* lines generated and used in this study are listed in Supplementary Table 1. Flies were reared under standard conditions. Oligos are listed in Supplementary Table 2. For quantification of fluorescent images and western blots, *p*-values were calculated using the two-tailed *T*-test for two independent means. For retinal degeneration assays, *p* values were calculated through Log-rank analysis using the Mantel-Cox test.

### eIF2D mutants (Df(3R)eIF2D)

To generate a Deficiency line deleting the *eIF2D* locus, we employed the FRT-mediated recombination strategy using two insertion lines PBac{PB}CG14512[c06309] and PBac{WH}eIF2D[f04182]. *eIF2D* rescue transgenes (depicted in Supplementary Fig. 1a), were amplified using a BACPAC plasmid (CH321-74G8) as template. For the *eIF2D*^*D109A*^ rescue transgene, the WT rescue plasmid was cut with unique sites Bsu36I and StuI and replaced with a corresponding D109A mutation-bearing DNA fragment generated by gene synthesis. The rescue fragments were cloned into the EcoR1 site in p-attB by InFusion assembly (ClonTech) and injected into embryos for targeted insertion into the attP2 locus. The resulting transgenes were recombined with the *Df(3R)eIF2D* lines to generate *Df.eIF2D*^*WT*^ wild type and the *Df.ΔeIF2D* mutant lines. To generate *eIF2D*^*CR1*^, we used CRISPR-Cas9 to introduce a single cut in the first exon of eIF2D and used homology directed repair to introduce a 22 bp deletion (see Supplementary Table 2 for guide RNA and homology arm primers).

*UAS-crc*^*leaderless*^ transgenic fly: The main ORF from the crc RA isoform was amplified from *w*^*1118*^ adult cDNA and was cloned into the pUAST plasmid between the EcoRI and XbaI sites. Transgenic flies were generated by injecting this plasmid in *w*^*1118*^ embryos by standard P-element transformation.

### Lethal phase analysis

50 L1 larvae of the appropriate genotype were picked and placed in a vial containing standard cornmeal molasses media supplemented with yeast paste. Three vials were tested per genotype for a total *n* of 150. Animals were allowed to mature at 25 °C for 14 days. After 14 days, animals that had not eclosed were transferred to a grape agar plate and allowed to age for an additional 7 days at 25 °C to determine final lethal phase. Bainbridge and Bownes staging criteria was used to determine lethal phases during metamorphosis^60^.

### Pupa imaging

Animals of the appropriate genotype and developmental stage were imaged while in the pupal case (for puparium morphology) or dissected out of the pupal case (for pupa morphology). All pupa images were taken on an Olympus SXZ16 stereomicroscope coupled to an Olympus DP72 digital camera with DP2-BSW software.

### Starvation assay

Second instar larvae of each genotype were moved to a new vial containing 4% sucrose as a nutrient source. The number of surviving larvae was counted 8 h later. *4E-BP* and *ATF4* mRNA levels in surviving larvae were assessed using qPCR.

### Retinal degeneration

The assays were done in the *cn.bw*^*−*/*−*^ background to eliminate eye pigments. The integrity of the photoreceptors was assessed through green fluorescent pseudopupils as seen by the *Rh1-GFP* transgene. Live flies for each genotype were monitored over 30 days and data were analyzed by log-rank test using the Kaplan–Meier method to determine significance.

### Structure-based identification of the D109 mutation

Analysis of the contact interface between RNA and PUA domains from several crystallographic structures (PDB 3LWP, 3LWR, and 3HAX), as well as the lower resolution cryoEM structure of human eIF2D bound to tRNA (5OA3) identified over ten PUA amino acid side chains contacting tRNA. We prioritized human D111 since the equivalent amino acid in the PUA domain of the human DKC1 protein has an established human genetic phenotype of causing the disease dyskeratosis congenita, reasoning that this established bioactivity in vivo would make it more likely to have a tRNA-specific expression phenotype in our experimental system. In order to confirm the 3D structural equivalence of *Drosophila* eIF2D D109 with human eIF2D D111, homology models of the *Drosophila* eIF2D PUA domain were made using previously described approaches^61^ using all of the PUA crystallographic and cryoEM templates and this amino acid side chain superimposed to observe that human D111 and *Drosophila* D109 occupied the same location in the structure.

### Immunostaining

Fat bodies or eye disks from wandering 3rd instar larvae were dissected in 1× PBS and fixed in 4% PFA, 1× PBS for 20 min. Tissues were then washed 1x with 1× PBS and permeabilized in 0.2% Triton-X 100, 1× PBS (PBST) for 20 min. The tissues were stained in PBST with primary antibodies at the indicated dilution and Alexafluor-conjugated secondary antibodies at 1:500 for 1.5 h, washed 3× with PBST and mounted with DAPI. All incubations were carried out at room temperature with mild agitation. Primary antibodies: Rabbit anti-GFP (1:500, Life Technologies A6455), Rabbit anti-RFP (1:250, Thermo Fisher R10367), Guinea pig anti-ATF4 (1:10^54^), Mouse anti-Rh1 (1:500, DSHB 4C5), Rabbit anti-PeIF2α (1:500, Cell Signaling 9721S).

### Western blotting

Cell lysates were prepared in 1% NP-40, 1× TBS on ice for 10 min and debris was cleared by centrifuging at 16,000×*g* for 10 min, 4 °C. Ten larval heads were dissected in 1× PBS and lysed similarly in 100 μl buffer containing 10 mM Tris-HCl [pH 7.5], 150 mM NaCl, protease inhibitor cocktail [Roche], 1 mM EDTA, 0.1% SDS. Lysates were analyzed on denaturing SDS-PAGE followed by western blotting with primary antibodies at indicated dilutions at 4 °C overnight and HRP-conjugated secondary antibodies (1:1000, Jackson Immunolabs) for 1 h at room temperature.

Primary antibodies: Guinea pig anti-eIF2D (1:500, raised against GST-tagged full length recombinant *Drosophila* eIF2D expressed using pet23a vector), Guinea Pig anti-Drosophila ATF4^24^, Rabbit anti-ATF4 (1:200, SCBT sc-200), Rabbit anti-PeIF2α (1:500, Abcam ab32157), Rabbit anti-eIF2α (1:2000, Abcam ab26197), Mouse anti-actin (1:5000, Millipore), Rabbit anti-GFP (1:1000, Life Technologies A6455).

### Immunoprecipitation assays

Larval lysates were prepared from 25 larvae as described above in RNAse-free lysis buffer containing 20U/ml of SUPERase-In RNAse inhibitor (Thermo Fisher). The lysates were incubated with anti-eIF2D antibody (1:100) for 2 h at 4 °C on an end-over-end rotator. 10 μl of Protein A Dynabeads (Thermo Fisher, prewashed 3× with lysis buffer) were added to the antibody:lysate mixture and incubated further for 2 h at 4 °C. Beads were washed with 1 ml ice-cold lysis buffer and eluted in 20 μl 1% SDS by boiling at 95 °C for 5 min. Eluates were analyzed on a 15% UREA-TBE gel for nucleic acid species.

For the nuclease-sensitivity assay, eluates were diluted tenfold in distilled water and DNase buffer (provided with TURBO DNase kit, Thermofisher). and digested with 1 μl TURBO DNase or RNase I (NEB) for 1 h at 37 °C. Digested eluates were analyzed on 15% UREA-TBE gel.

### Cell culture

WT and ΔeIF2D HAP1 cells (Horizon Discovery, HZGHC002651c012) were cultured according to supplier’s protocol. Cells were transfected with negative control scrambled siRNA (Qiagen) or *DENR* siRNA (Dharmacon, L-012614-00-0005) using Lipofectamine 2000 (Life Technologies) according to manufacturer’s protocol. Human *eIF2D* (Dharmacon, 4339127) was subcloned into the EcoRI and XbaI sites of pcDNA3.1 and cotransfected with siRNA.

### Reporting summary

Further information on research design is available in the Nature Research Reporting Summary linked to this article.

## Supplementary information

Supplementary Information

Reporting Summary

## Data Availability

The data that support the findings of this study are available from the corresponding author upon reasonable request. All fluorescent microscopy images (Figs. 1b–e, 2c, 3b, 4c–f and Supplementary Figs. 1c, 2a, 4a, 5) and confocal microscopy images (Figs. 1f–i, 2d–i, 3c–j, 6a–i and Supplementary Figs. 1d, e, 2b–e, 4b–d, 6b, c, 7a–c) presented in the manuscript have associated raw images, and are available from the corresponding author upon reasonable request. Source data are provided with this paper.

## Source data

Source Data
