# Supplementary material for: Translational induction of ATF4 during integrated stress response requires noncanonical initiation factors eIF2D and DENR
Source: Nat Commun. 2020 Sep 16;11:4677. doi: 10.1038/s41467-020-18453-1 (PMC7495428; doi:10.1038/s41467-020-18453-1)
Supplement: Supplementary file 1 — Supplementary Information [file 41467_2020_18453_MOESM1_ESM.pdf]

Translational induction of ATF4 during Integrated Stress Response requires noncanonical initiation factors eIF2D and DENR

Vasudevan et al.

Supplementary Information

Supplementary discussion

Extended analysis of lethal phase and animal morphology

As depicted in Fig. 4a, two molts occur during larval development. Puparium formation marks the onset of metamorphosis and is the developmental transition from larval development to prepupal development. Pupation marks the transition from prepupal development to pupal development, and eclosion is the emergence of an adult fly. As shown in Fig. 4b, all control animals survive to adulthood, as do the majority of *eIF2D* mutant animals. Nearly all *DENR* mutant animals arrest development after pupation. A significant percentage of *crc* and *DENR eIF2D* double mutant animals arrest development as larvae, likely due to defects in molting. The remaining *crc* and *DENR eIF2D* double mutant animals arrest during metamorphosis, either as prepupae or as cryptocephalic pupae (cryptocephalic pupae indicated by the star symbol).

The pupal case, or puparium, provides a phenotypic readout of the morphogenetic events that occur during puparium formation. *eIF2D* (Supplementary Fig. 5b) and *DENR* mutant animals (Supplementary Fig. 5c) form normal puparia that are similar to controls (Supplementary Fig. 5a). As shown in the lethal phase graph (Fig. 4b), a substantial percentage of *crc* and *DENR eIF2D* double mutant animals arrest development as prepupae. These animals exhibit severe defects in puparium formation, including failure to properly harden the cuticle (shown by a ridged appearance to the puparium), impaired body contraction (evidenced by the lack of barrel shape), and failure to evert the anterior spiracles (*c.f.* Supplementary Fig. 5a vs. d and e).

The images in Supplementary Fig. 5 also illustrate morphological defects of animals inside the pupal case. *DENR* mutant animals (Supplementary Fig. 5c) exhibit defects in pupation, including failure to properly evert the head and extend the wings and legs (solid white arrowheads and outlined white arrowheads, respectively). These defects are more easily discernible in dissected pupae (Fig. 4c-f). *Canton S* control animals (Fig. 4c) display the normal extent of head eversion and wing/leg extension. *crc* mutant animals (Fig. 4f) display a complete failure in pupation, evidenced by total absence of a head (cryptocephaly) and complete failure to extend the wings and legs. These animals also fail to properly contract the abdomen at puparium formation, resulting in an elongated body axis. However, *crc* mutant animals can deposit cuticle and develop pigmentation in the wings and legs. *DENR* mutant animals (Fig. 4d) display defects indicative of partial/incomplete pupation, including failure to fully evert the head and failure to fully extend the wings and legs. The additional loss of one copy of *eIF2D* (*DENR*^{KO} *eIF2D*^{CR1}*/TM3*) (Fig. 4e) results in complete failure of pupation, resulting in a strong cryptocephalic phenotype that closely resembles *crc* mutant animals.

Supplementary Fig. 1

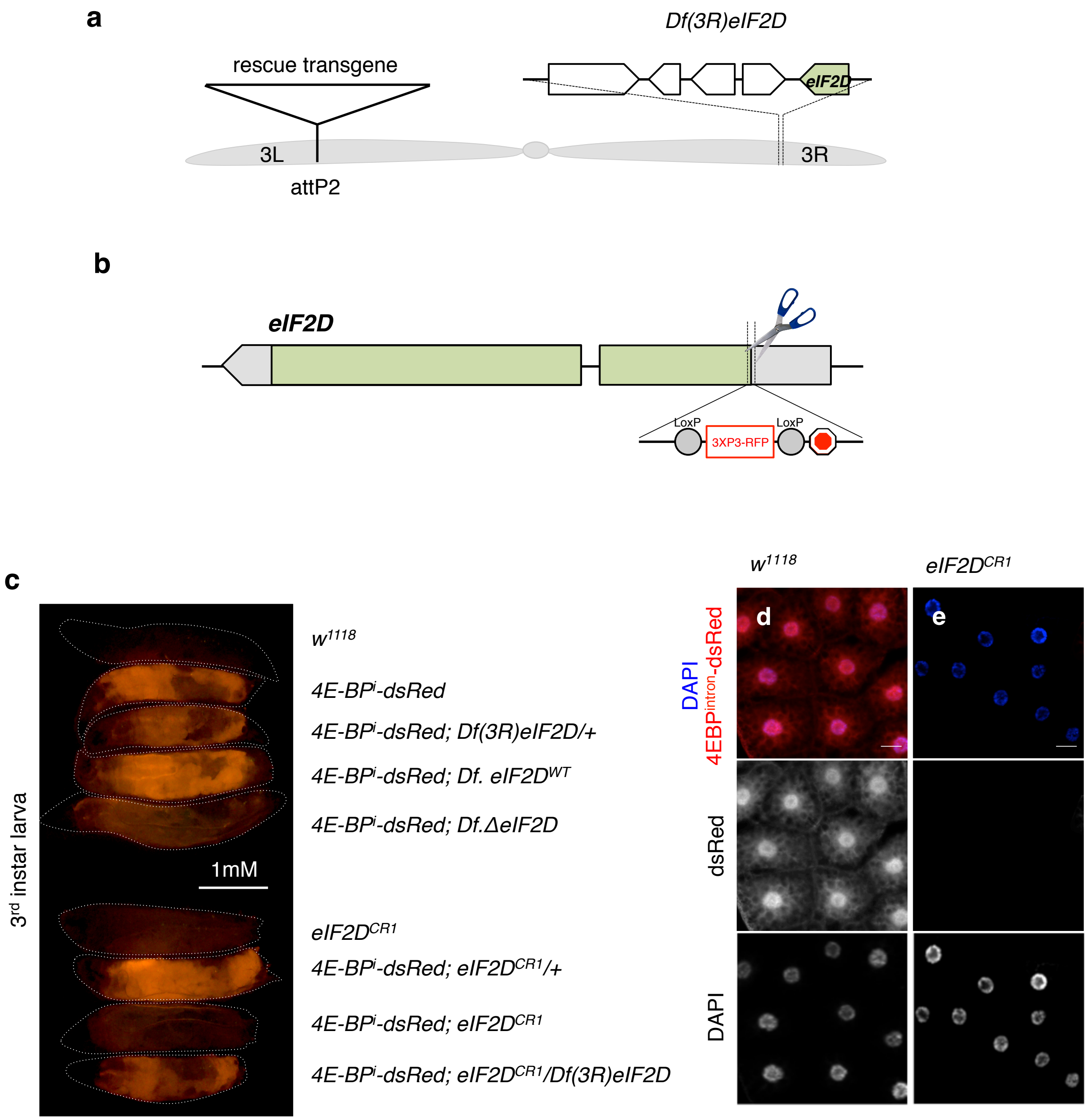

Supplementary Figure 1. Generation and validation of *eIF2D* mutants.

(a, b) Genomic locus of *eIF2D* on chromosome 3R showing the schematic for generating *eIF2D* mutants. A large deficiency (*Df(3R)eIF2D*) was created by FRT-mediated deletion of the region between two PBac insertions spanning *eIF2D* (indicated in green). The deficiency was complemented by inserting genomic rescue constructs (containing either wild type or *eIF2D* mutant sequence) spanning the indicated regions in the same attP2 landing site on 3L. The $eIF2D^{CR1}$ allele was engineered with CRISPR-Cas9 using a single guide RNA targeting the 5' UTR of *eIF2D* and selected by homology-mediated insertion of a RFP marker.

(c) dsRed expression in control larvae (w^{1118}) and larvae bearing $4E\text{-}BP^{intron}$-dsRed in homozygous and trans-heterozygous *eIF2D* mutants. Individual larvae are outlined with dotted lines.

(d, e) $4E\text{-}BP^{intron}$-dsRed expression (red) in fat bodies of control and homozygous $eIF2D^{CR1}$ mutant larvae. DAPI (blue) marks nuclei.

Data in (c-e) are representative images collected from two biologically independent experiments with 10 animals in each trial.

Supplementary Fig. 2

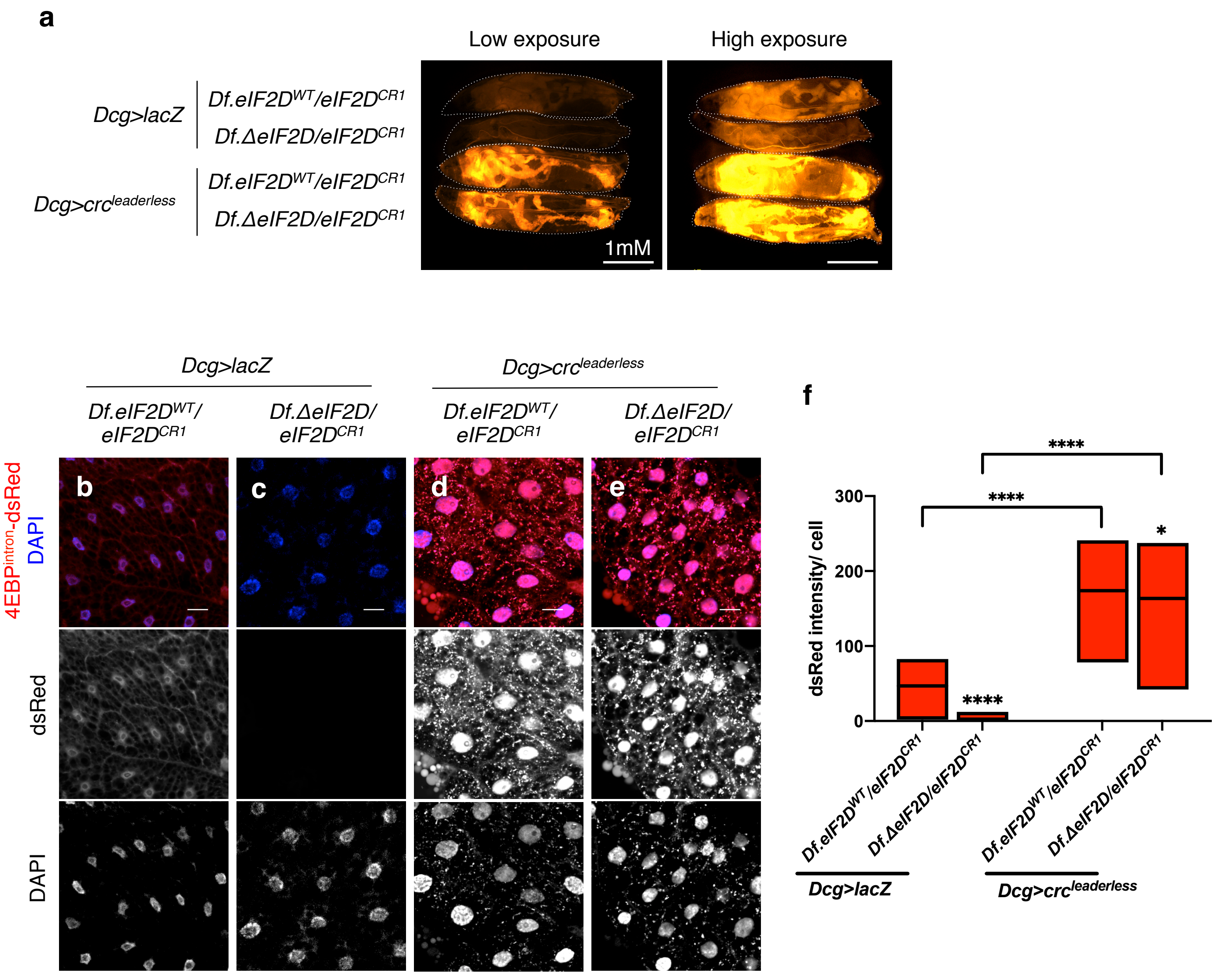

Supplementary Figure 2. Rescue of 4E-BP^{intron}-dsRed expression in eIF2D mutants by ectopic expression of transgenic *crc* (*Drosophila ATF4*).

(a) dsRed expression in control and *eIF2D* transheterozygous mutant larvae bearing 4E-BP^{intron}-dsRed, expressing either a control rescue transgene (lacZ) or *crc*^{leaderless} (*crc* without its 5' leader) driven by *Dcg-GAL4.* Individual larvae are outlined with dotted lines. Two different exposure times are shown to highlight the differences between the genotypes. Note that overexpression of *crc* increases dsRed levels in control and mutant larvae to higher than normal levels seen during development (*c.f* higher exposure panel and Supplementary Fig. 1c for example), and causes a 'stringy' appearance of the fat tissues (seen in the lower exposure panel).

(b-e) 4E-BP^{intron}-dsRed expression (red) in fat bodies dissected from larvae in (a), counterstained with DAPI (blue).

Data in (a-e) are representative images collected from two biologically independent experiments with 5 animals in each trial.

(f) Quantitation of dsRed intensity from individual cells in (b-e). Midline represents the mean value, with the top and bottom of the box representing the maximum and minimum values. Asterisks above boxes represent statistical significances between mutant and control values calculated with the a two-tailed t-test with ****= $p<0.00001$ and *= $p<0.01$. n= 152, 173, 177 and 141 respectively for each box from left to right. Please see Source Data Files for raw data.

Supplementary Fig. 3

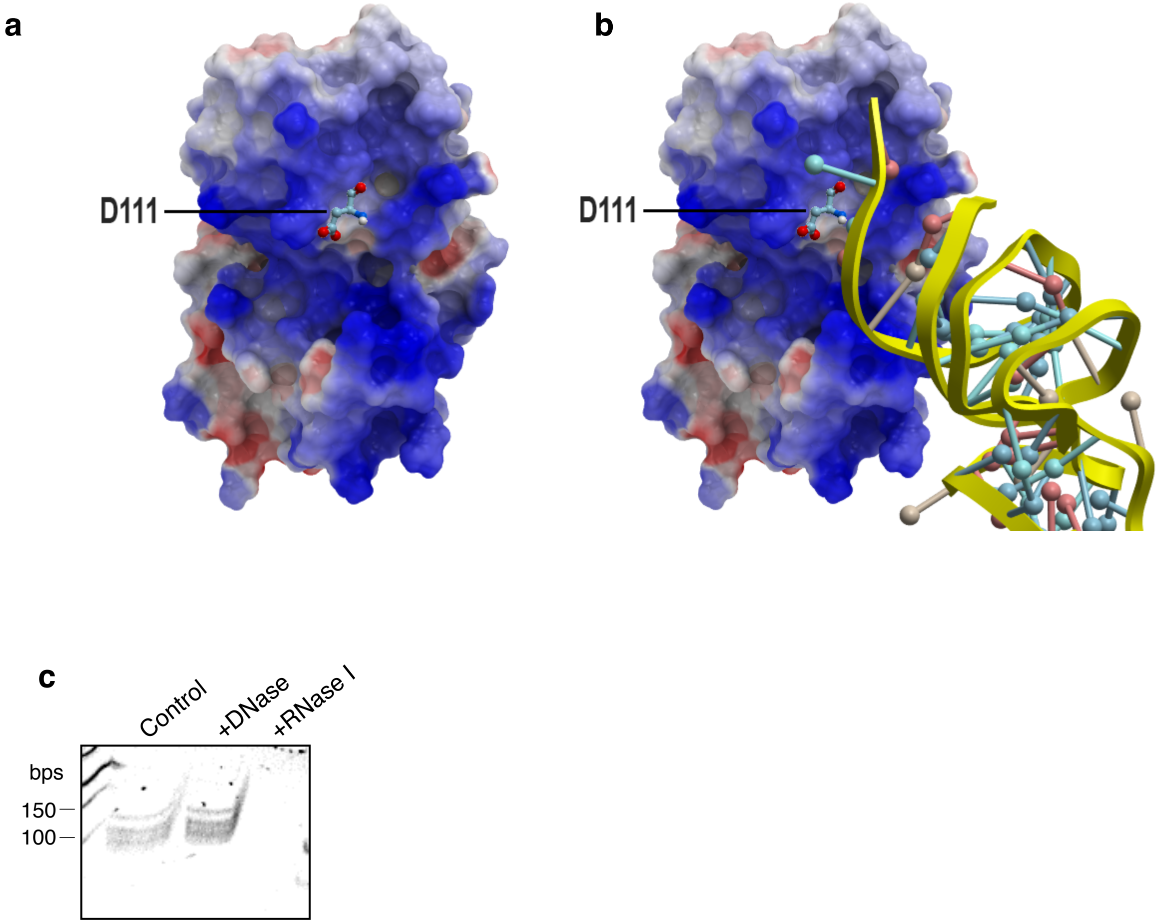

Supplementary Figure 3. An eIF2D amino acid residue at the tRNA-binding interface.

(a, b) The position of D111 residue in Human eIF2D structure (based on PDB: 5oa3). Negatively charged protein surface is colored red, and positively charged areas in blue. The structures show a surface in eIF2D with (a), or without the bound tRNA (b).

(c) UREA-TBE gel analysis of nucleic acid species that was coimmunoprecipitated with wild type eIF2D in 2k after treatment with DNase or RNase I. Data is a representative image from two biologically independent experiments. Please see Source Data Files for raw data.

Supplementary Fig. 4

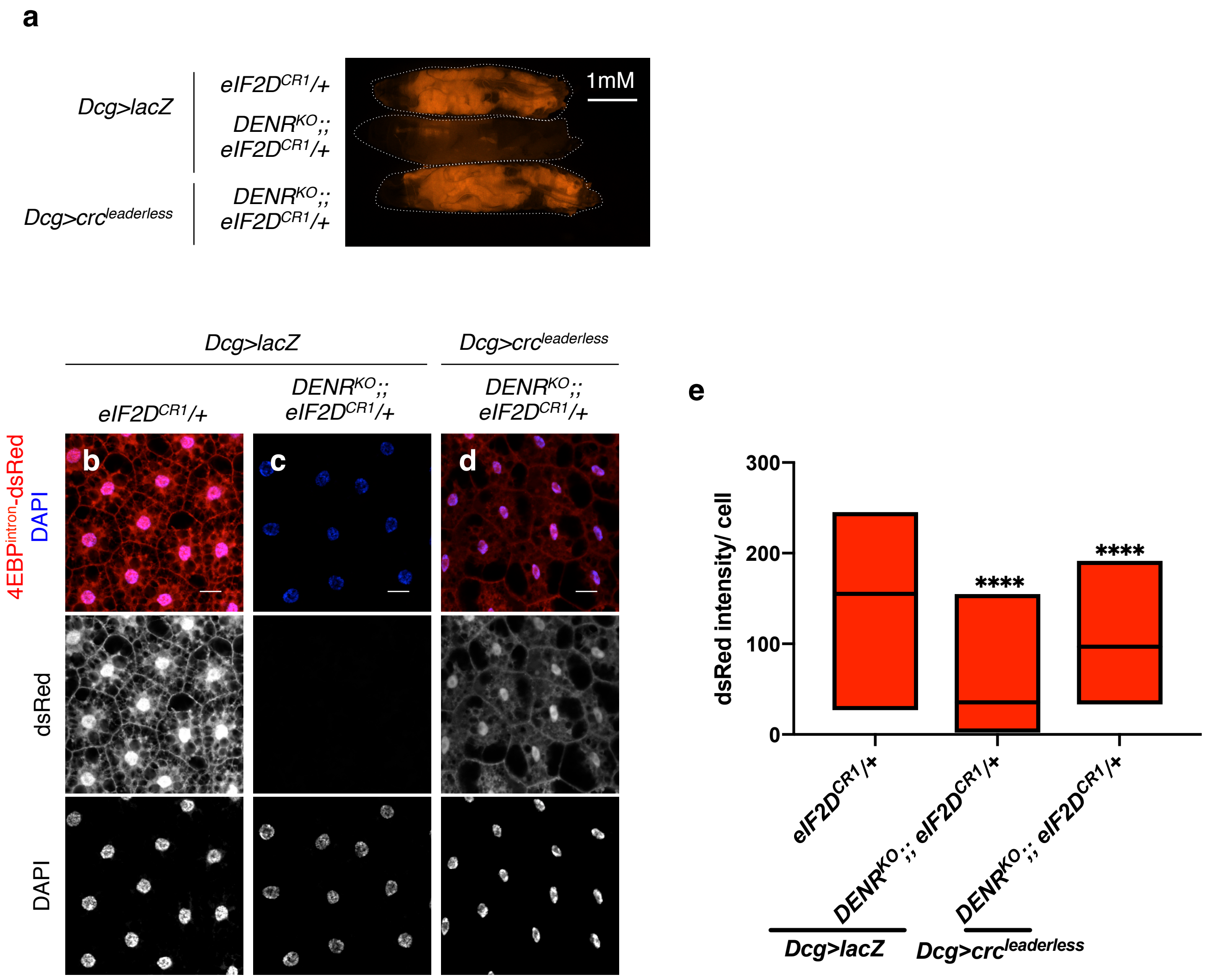

Supplementary Figure 4. Rescue of 4E-BP^{intron}-dsRed in eIF2D and DENR mutants with ATF4

(a) 4E-BP^{intron}-dsRed expression in control and mutant larvae where *Dcg-GAL4* drives expression of a control transgene (*lacZ*) or *crc*^{leaderless}. Individual larvae are outlined with dotted lines. Note that these animals were raised at 20°C.

(b-d) Fat body tissues dissected from larvae in (a) showing 4E-BP^{intron}-dsRed expression (red), and counterstained with DAPI (blue).

Data in (a-d) are representative images collected from two biologically independent experiments with 4 animals in each trial.

(e) Quantitation of dsRed intensity from individual cells in (b-d). Data were collected from 206-250 cells from 5 animals. Midline represents the mean value, with the top and bottom of the box representing the maximum and minimum values. Asterisks above boxes represent statistical significances between mutant and control values calculated with the a two-tailed t-test with ****= $p<0.00001$. n= 206, 250 and 247 respectively for each box from left to right.

Supplementary Fig. 5

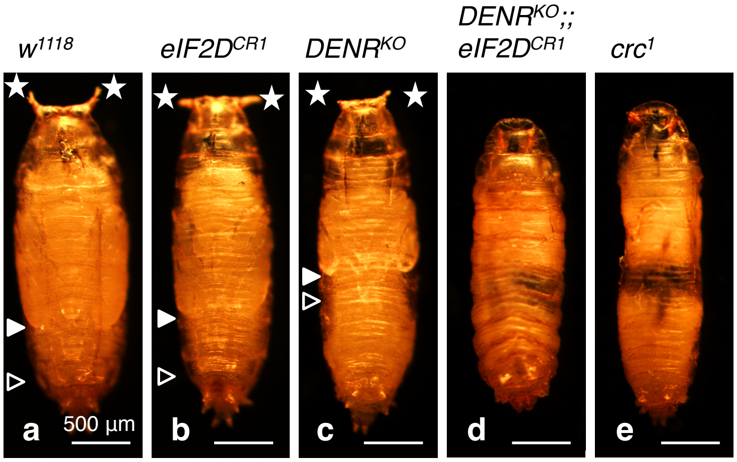

Supplementary Figure 5. Analysis of *eIF2D* and *DENR* mutant phenotypes.

(a-e) Analysis of puparium (pupal case) morphology in control, *eIF2D*, *DENR*, *DENR eIF2D* double, and *crc* mutant animals. Stars mark the presence of the anterior spiracles present in a normal puparium. Solid white arrowheads indicate the degree of wing extension and outlined white arrowheads indicate the degree of leg extension. Note that no wing or leg structures are discernible in *crc* or *DENR eIF2D* double mutant animals. Data are representative images collected from two biologically independent experiments with 15 animals in each trial.

Supplementary Fig. 6

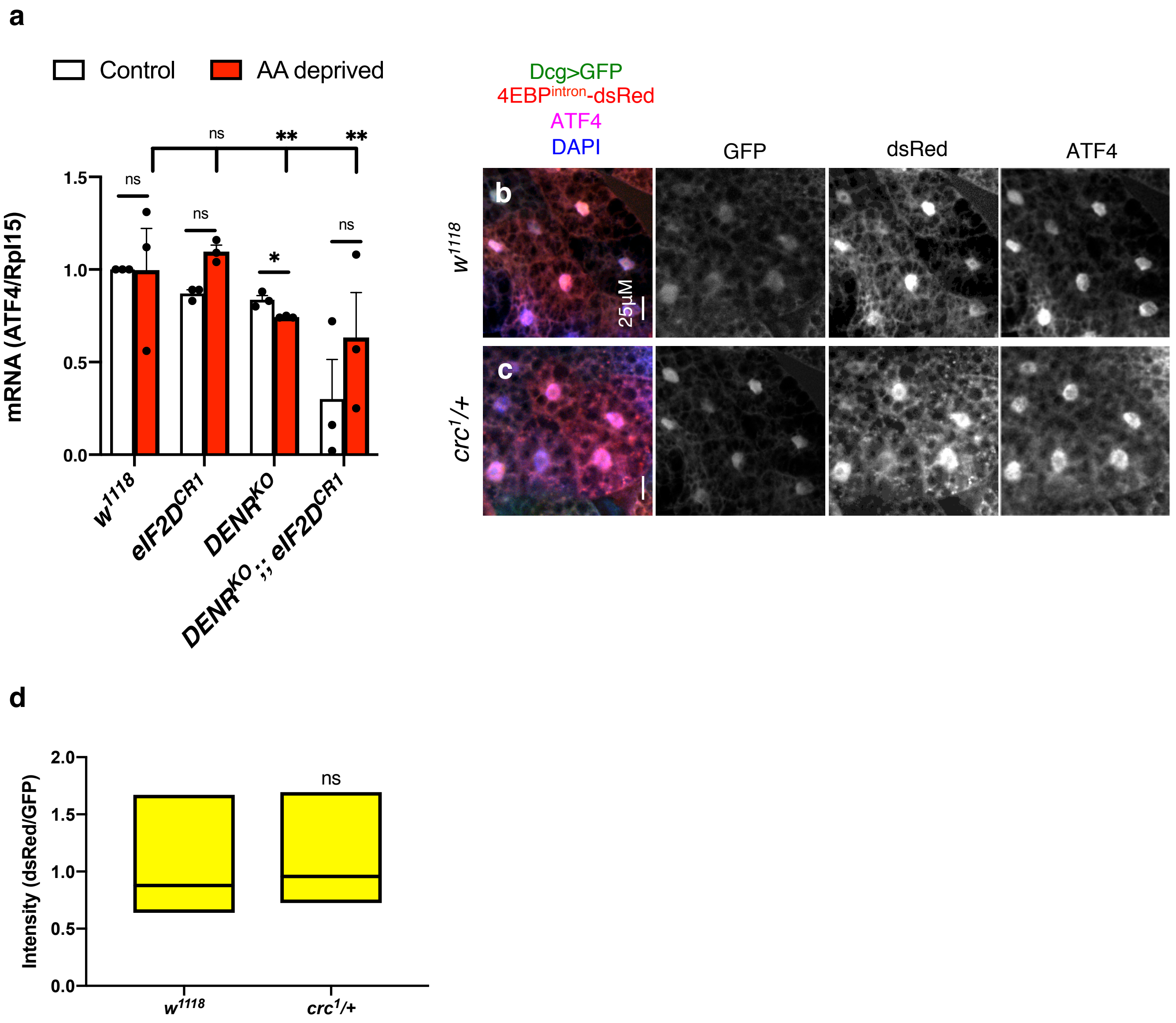

Supplementary Figure 6. *ATF4* mRNA levels may be affected in DENR eIF2D double mutants

(a) qPCR analysis of ATF4 mRNA from larvae in Fig 5a. Data represent the mean from three independent experiments. Error bars represent standard error, p values were calculated using the two-tailed t-test with *=p<0.01, **= p<0.001, and n.s. = not significant.

(b, c) 4E-BP^{intron}-dsRed expression in fat bodies of control and heterozygous crc^1 larvae. Data are representative images collected from one biological experiment with 15 animals.

(d) Quantification of the 4E-BP^{intron}-dsRed reporter intensities normalized to corresponding GFP intensities from the same cells in (b, c). Midline represents the mean value, with the top and bottom of the box representing the maximum and minimum values. n.s=not significant. n= 42 ad 48 respectively for each box from left to right.

Please see Source Data Files for raw data in (a, d).

Supplementary Fig. 7

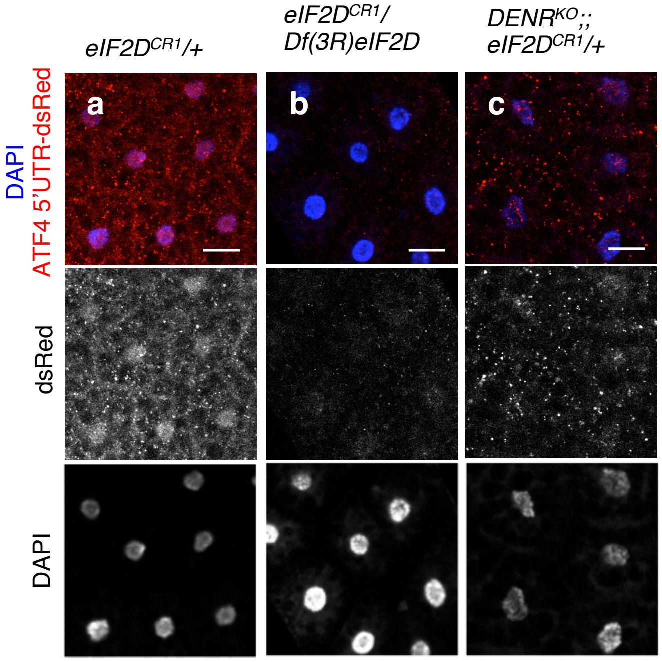

Supplementary Figure 7. *eIF2D* and *DENR* regulate ATF4 translation via it's 5' leader

(a-c) Expression of the ATF4 5'UTR-dsRed reporter in fat bodies of *eIF2D* transheterozygous mutant, $DENR^{KO}$ mutant and respective control larvae. Data are representative images collected from three biologically independent experiments with 10 animals in each trial.

Supplementary Fig. 8

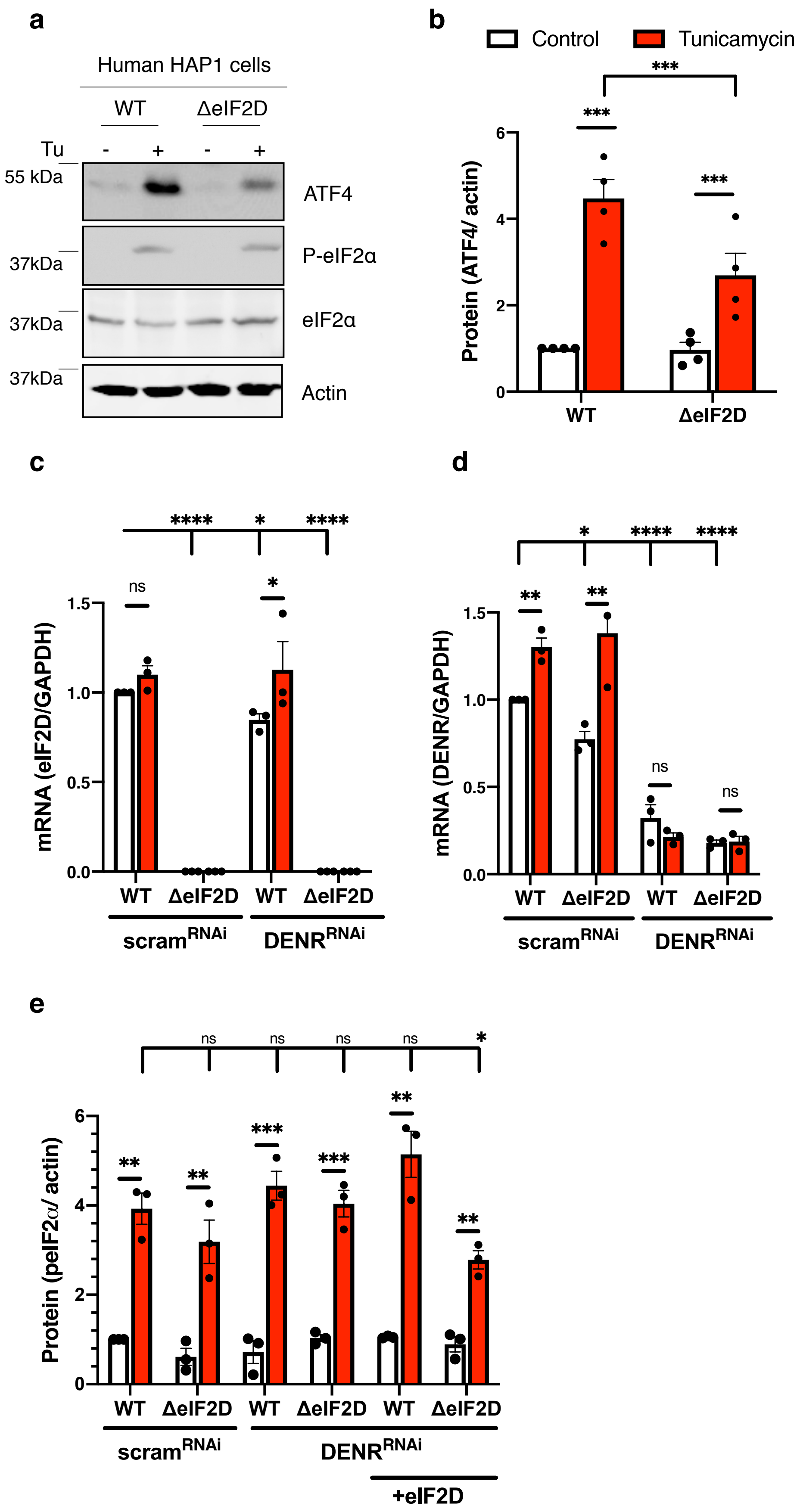

Supplementary Figure 8. Characterization of *eIF2D* and *DENR* in human cells.

(a) Western blots from WT and ΔeIF2D HAP1 human cells with or without Tunicamycin (Tu) treatment.

(b) Quantification of western blot from *WT* and *ΔeIF2D* HAP1 cells from (a) with data representing the mean of 4 different experiments.

(c-d) qPCR analysis of eIF2D (b) and DENR (d) in HAP1 cells from Fig. 7a normalized to GAPDH. Data represent the mean of 3 different experiments.

(e) Quantitation of peIF2α protein levels in (Fig. 7a) as normalized to the loading control (actin). Data are the mean of 3 independent experiments.

Error bars in all panels represent standard error, p values were calculated using the two-tailed t-test with *=p<0.01, **= p<0.001, ***=p<0.0001, =p<0.00001 and n.s. = not significant.

Please see Source Data Files for raw data in (a-e).

Supplementary Fig. 9

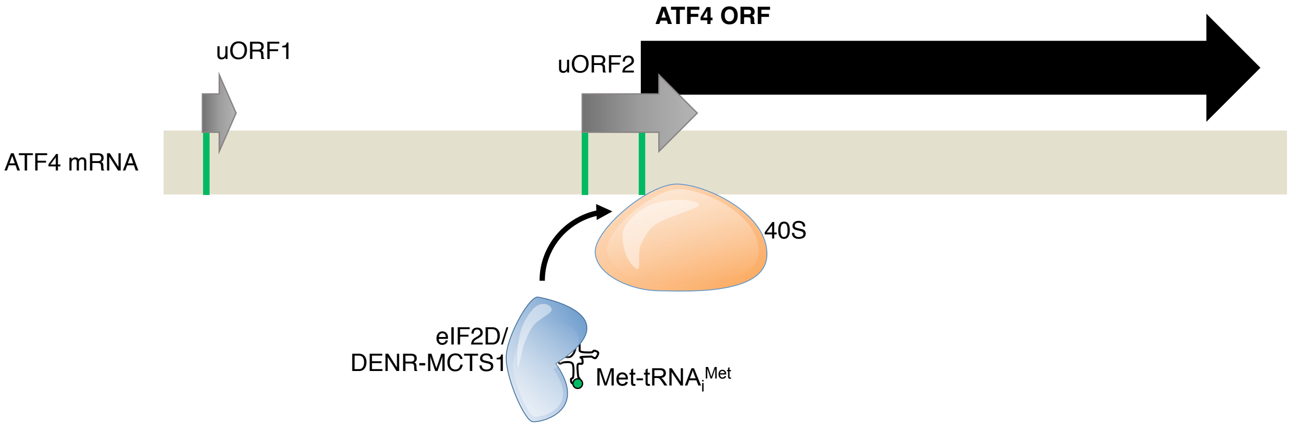

Supplementary Figure 9. A model for the function of eIF2D and DENR

(a) A schematic shows the structure of human ATF4 mRNA with uORFs 1, 2 (gray arrows) and the main ATF4 ORF (black arrow). Green bars represent AUG start codons for each ORF. eIF2D or DENR-MCTS1 (in blue) acts as non-canonical Met-$tRNA_i^{Met}$ (black outline, green circle) recruiters to the ATF4 ORF along with the 40S ribosome subunit (orange).

Supplementary Figure 9. A model for the function of eIF2D and DENR

(a) A schematic shows the structure of human ATF4 mRNA with uORFs 1, 2 (gray arrows) and the main ATF4 ORF (black arrow). Green bars represent AUG start codons for each ORF. eIF2D or DENR-MCTS1 (in blue) acts as non-canonical Met-tRNA$_i^{Met}$ (black outline, green circle) recruiters to the ATF4 ORF along with the 40S ribosome subunit (orange).

Supplementary tables

Supplementary Table 1. List of fly stocks used.

Fly stocks were sourced from the Bloomington Drosophila Stock Center (BDSC), Harvard Exelixis (Harvard) and the Vienna Drosophila RNAi Center (VDRC). RNAi lines targeting annotated translational initiation factors were selected for the screen in addition to positive control RNAi lines targeting *ATF4* and *GCN2*, which were previously shown to suppress 4E-BP^{intron}-dsRed reporter expression[24]. Also included were *FOXO* RNAi lines that do not affect the dsRed reporter.

**Genotype**	**Source**	**Stock number**
4E-BP^{intron}-dsRed	[24]	
Dcg-Gal4	[24]	
ATF4 5'UTR-dsRed	[54]	
PBac{PB}$CG14512^{c06309}$	Harvard	c06309
PBac{WH}$eIF2D^{f04182}$	BDSC	18740
$DENR^{KO}$	BDSC	76341
cn^1 crc^1/SM5	BDSC	266
GMR-Gal4	BDSC	1104
ey-FLP	BDSC	5580
UAS-$Rh1^{G69D}$	[50]	
$Perk^{e01744}$	Harvard	e01744
$ninaE^{G69D}$	[50]	

**RNAi lines used in screen**		
**Target gene**	**VDRC stock number**	**<u>Effect on Dcg>GFP and 4E-BP^{intron}-dsRed</u>**
CG1442 <u>(eIF4E6)</u>	17580	No change
	17581	No change

CG2238 (Ef2b)	107268	Developmentally arrested before 3rd instar
CG2677 (eIF2Bbeta)	25426	GFP and dsRed very low
	25427	
CG3143 (foxo)	106097	No change
	107786	
CG3186 (eIF5A)	101513	Small decrease in dsRed, no change in GFP
CG3806 (eIF2B-ε)	34711	Developmentally arrested before 3rd instar
CG4035 (eIF4E)	100722	No change
	7800	
CG4153 (eIF2β)	105291	Developmentally arrested before 3rd instar
	48911	
CG4878 (eIF3-S9)	107829	Developmentally arrested before 3rd instar
	27609	
CG4954 (eIF3-S8)	26664	Developmentally arrested before 3rd instar
	26667	No change
CG7483 (eIF4A-III)	108580	No change
CG8053 (eIF1A)	100611	Developmentally arrested before 3rd instar
	26022	No change in GFP, increased dsRed
CG8190 (eIF2Bγ)	108083	Developmentally arrested before 3rd instar
	43917	
CG8277 (eIF4E5)	102173	No change in GFP, decrease in dsRed
	24267	No change
CG9075 (eIF4A)	1000310	Developmentally arrested before 3rd instar
	42202	
CG9099 (DENR)	101746	No change
	49895	

CG9124 eIF3p40	106189	No change in GFP, small decrease in dsRed
	36087	
CG9946 eIF2alpha	104562	Developmentally arrested before 3rd instar
	7799	
CG9769 eIF3-S5	101465	No change in GFP, increase in dsRed
CG10315 eIF2Bδ	104403	Developmentally arrested before 3rd instar
	48708	
CG10811 eIF4G	17002	Developmentally arrested before 3rd instar
	17003	
CG10840 eIF5B	109782	Developmentally arrested before 3rd instar
	31366	No change
CG10837 eIF4B	31364	No change
CG10990 Pdcd4	16160	No change
	16162	No change in GFP, decrease in dsRed
CG17737 eIF1	105763	Developmentally arrested before 3rd instar
	29216	No change
CG17611 eIF6	108094	No change in GFP, small decrease in dsRed
CG31426 eIF2D	100304	No change in GFP, modest decrease in dsRed
	29216	No change in GFP, strong decrease in dsRed
CG9805 eIF3-S10	21840	No change
CG43665 eIF2γ	33834	No change
CG1609 GCN2	103976	No change in GFP, strong decrease in dsRed
CG8669 ATF4	2934	Developmentally arrested before 3rd instar
	2935	

	109014	No change in GFP, strong decrease in dsRed
CG2087 (PERK)	110278	No change in GFP, small decrease in dsRed

Supplementary Table 2. List of oligos used.

Primer, figure	Sequence
eIF2D^{WT} rescue	F: CTGAATAGGGAATTGGGGGCTAACCCCTTGGGGTTGGCACCAAAAG<br>R: CAGATCTGTTAACGAATTGTTTTGTTGAATAAAATGGCGTCTTAC
∆eIF2D rescue	F: CTGAATAGGGAATTGGGGGCTAACCCCTTGGGGTTGGCACCAAAAG<br>R: CAGATCTGTTAACGAATTTGAAGCCTCCTCCGCCTGTCCTCTTGAGTGT
eIF2D^{D109A} fragment	AGGCCTTTGTCTTGACCAGCGGGATTTGCTGCACCAGACTGGGCTCATGCGA<br>CCAGAGCTTGTCCCCAAAGAGGTGGAGCATCTTGATGGCCACACCGTGACCT<br>CCGCACATGTACAGTTCATCGCTGGAGCGAGCCAACTGGCCCACGCCCACCG<br>CAGATTTGTTGTTTGTGAGGTTTACGGCCACTGAAAAAGACAATGAGATCAA<br>TACTCGTTACCTGGGGAATCCCAAGCAGATACCCACTCAACTGTCCCTTTTT<br>GAAGTGCCCGTACATGCTCAGTCCCACGCCCAGGGGCACCACTCCGGGCAGC<br>ATGAGAGCCGCTCCGTTCGTCAGCTTGGGTAACACACCCTCGTGGGTGGTGA<br>AATAGGGCAGGATGTCCGGAACGATCCACAGGGTGTACAGGGTGGGCACAAG<br>TTGACCGCCGTCCAGCTCGAAGAACAAAGGCAACTTGTCCACGCAGTAGACC<br>ATGCTCTGAACACCGCCGTGCGTTAGGATCTTCACCTGGGTCACCGCCGCCT<br>TCGCCGGCACCAGCTGATCCACGGACACATGGGGGAACGCCGCCTCCACGCG<br>CTGCCGGAACTTCTTGCTATCGGATCCCTTGAGGGCAGCACTGCTCTTGGGT<br>CTGTACGGCTTGAGAAACATCTGTGTTTGGTGGTGTGCACTTGAGGAGTTCG<br>TGAAATGGTTTGGAGTGGCACATGCACAGTGAAAACAAACAACTGGCAAACG<br>GGGGTGCTGCTTTAGCTATTTTCTCGCCAGTTTGTATATTTAAAAAAATATC<br>AATGGGATTCGATATTCTAAAACCAAGTATAAGAAACATAAGAATAAGATGC<br>AGAAAAAGAAATTAACATAAAATAAAGAAGTGAGTCCTGTCTTTTCTTTTCA

	CAATAACTCTACCCTAAATCAAAATTAGTTAAGAGCAGACGTTATTACAAGG ACAGCACGCGCAGTAAAAATGCCGGCGGTCCGGAAAATTTCGAATCACCAAG GACTTCCTCAGG
Guide RNA primers	Sense: CTTCGCAAGAGCAGTGCTGCCCTCA<br>Antisense: AAACTGAGGGCAGCACTGCTCTTGC
5' homology arm in eIF2D^{CR1}	F: CTCCAACAGTCCAAACAGCA<br>R: GGGTCTGTACGGCTTGAGAA
3' homology arm in eIF2D^{CR1}	F: GATCCGATAGCAAGAAGTTCC<br>R: ATCCACTCCCTTGCTCTCCT
Primers for crc-RA	F: ATCGGAATTCATGGACGCGACATTTCACCA<br>R: ATCGTCTAGACTAGCGCTTGCGTTCATGG
**qPCR primers**	
d4E-BP	F: TAAGATGTCCGCTTCACCCA<br>R: CGTAGATAAGTTTGGTGCCTCC
dATF4	F: GGAGTGGCTGTATGACGATAAC<br>R: CATCACTAAGCAACTGGAGAGAA
heIF2D	F: CCTTGGAACTGATCAAGTCTCTG<br>R: CATGTTGTAAAGGTTGGCAGAAG
hDENR	F: AAGATGTCTGTGTGCCTGTATC<br>R: CATCCACTGCTGGCTATCTATG
hATF4	F: GGAGATAGGAAGCCAGACTACA<br>R: GGCTCATACAGATGCCACTATC
