# Supplementary material for: Translational induction of ATF4 during integrated stress response requires noncanonical initiation factors eIF2D and DENR
Source: Nat Commun. 2020 Sep 16;11:4677. doi: 10.1038/s41467-020-18453-1 (PMC7495428; doi:10.1038/s41467-020-18453-1)
Supplement: Supplementary file 2 — Reporting Summary [file 41467_2020_18453_MOESM2_ESM.pdf]

Corresponding author(s): Hyung Don Ryoo

Last updated by author(s): Jul 14, 2020

Reporting Summary

Nature Research wishes to improve the reproducibility of the work that we publish. This form provides structure for consistency and transparency in reporting. For further information on Nature Research policies, see our Editorial Policies and the Editorial Policy Checklist.

Statistics

For all statistical analyses, confirm that the following items are present in the figure legend, table legend, main text, or Methods section.

n/a	Confirmed	
☐	☒	The exact sample size (n) for each experimental group/condition, given as a discrete number and unit of measurement
☐	☒	A statement on whether measurements were taken from distinct samples or whether the same sample was measured repeatedly
☐	☒	The statistical test(s) used AND whether they are one- or two-sided<br>*Only common tests should be described solely by name; describe more complex techniques in the Methods section.*
☒	☐	A description of all covariates tested
☒	☐	A description of any assumptions or corrections, such as tests of normality and adjustment for multiple comparisons
☐	☒	A full description of the statistical parameters including central tendency (e.g. means) or other basic estimates (e.g. regression coefficient) AND variation (e.g. standard deviation) or associated estimates of uncertainty (e.g. confidence intervals)
☐	☒	For null hypothesis testing, the test statistic (e.g. F, t, r) with confidence intervals, effect sizes, degrees of freedom and P value noted<br>*Give P values as exact values whenever suitable.*
☒	☐	For Bayesian analysis, information on the choice of priors and Markov chain Monte Carlo settings
☒	☐	For hierarchical and complex designs, identification of the appropriate level for tests and full reporting of outcomes
☒	☐	Estimates of effect sizes (e.g. Cohen's d, Pearson's r), indicating how they were calculated

Our web collection on statistics for biologists contains articles on many of the points above.

Software and code

Policy information about availability of computer code

Data collection	DP2-BSW software for Olympus DP72 camera, Zen 3.2 software on Zeiss LSM 700, Odyssey 3.0 software for LI-COR
Data analysis	ImageJ 1.49d for image analysis, Prism v8.0.2 (for graphing and statistical analysis)

For manuscripts utilizing custom algorithms or software that are central to the research but not yet described in published literature, software must be made available to editors and reviewers. We strongly encourage code deposition in a community repository (e.g. GitHub). See the Nature Research guidelines for submitting code & software for further information.

Data

Policy information about availability of data

All manuscripts must include a data availability statement. This statement should provide the following information, where applicable:

- Accession codes, unique identifiers, or web links for publicly available datasets
- A list of figures that have associated raw data
- A description of any restrictions on data availability

All fluorescent miscroscopy images (Figures 1b-e, 2c, 3b, 4c-f, Supplementary figures 1c, 2a, 4a, 5), confocal microscopy images (Figures 1f-i, 2d-i, 3c-j, 6a-i, Supplementary figures 1d-e, 2b-e, 4b-d, 6b-c, 7a-c) and gel images (Figures 2a, 2k, 7a, Supplementary figures 3c, 8a) presented in the manuscript have associated raw images. Source data for all gel images and graphing data is provided with the manuscript.

Field-specific reporting

Please select the one below that is the best fit for your research. If you are not sure, read the appropriate sections before making your selection.

☒ Life sciences ☐ Behavioural & social sciences ☐ Ecological, evolutionary & environmental sciences

For a reference copy of the document with all sections, see nature.com/documents/nr-reporting-summary-flat.pdf

Life sciences study design

All studies must disclose on these points even when the disclosure is negative.

Sample size	Sample sizes were based on the applicable standards in the field. For genetics experiments involving crossing animals, each cross was set up at least two times, and animals were randomly sampled across the experimental set ups.
Data exclusions	No data were excluded.
Replication	All experiments were performed 3 times, except Fig 3c which was performed 2 times, to generate biological replicates. For genetics experiments involving crossing animals, each cross was set up at least two times.
Randomization	All animals were of similar age and were assigned to control or experimental groups randomly. For experiments involving cell lines, cell culture dishes were randomly assigned to control and Tunicamycin-treated groups.
Blinding	The RNAi lines for the original screen was performed blind where the experimenter was unaware of the identity of the RNAi lines, and rated the fluorescence intensity for 10 randomly chosen animals. For all other experiments, blinding was not possible due to limited personnel availability.

Reporting for specific materials, systems and methods

We require information from authors about some types of materials, experimental systems and methods used in many studies. Here, indicate whether each material, system or method listed is relevant to your study. If you are not sure if a list item applies to your research, read the appropriate section before selecting a response.

Materials & experimental systems

n/a	Involved in the study	
☐	☒	Antibodies
☐	☒	Eukaryotic cell lines
☒	☐	Palaeontology and archaeology
☐	☒	Animals and other organisms
☒	☐	Human research participants
☒	☐	Clinical data
☒	☐	Dual use research of concern

Methods

n/a	Involved in the study	
☒	☐	ChIP-seq
☒	☐	Flow cytometry
☐	☐	MRI-based neuroimaging

Antibodies

Antibodies used	Primary antibodies: Guinea pig anti-eIF2D (raised against GST-tagged full length recombinant Drosophila eIF2D expressed using pet23a vector) and Guinea Pig anti-Drosophila ATF4 (Kang et al, PMC4433282) were made in our laboratory. The following commercially available antibodies were also used: Rabbit anti-RFP (ThermoFisher, #R10367) Rabbit anti-ATF4 (Santa Cruz Biotechnology, #sc-200), Rabbit anti-PeIF2alpha(AbCam, #ab32157), Rabbit anti-eIF2alpha(AbCam, #ab26197), Mouse anti-actin (Millipore, #MAB1501), Rabbit anti-GFP (Life Technologies, #A6455).<br><br>Secondary antibodies: The following Alexa fluor conjugated secondaries from Life Technologies were used for immunofluorescence- Goat anti-Guinea Pig Alexa fluor 647 (#A-21450), Goat anti-Rabbit Alexa fluor 488 (#A-11008), Goat anti-Rabbit Alexa fluor 546 (#A-11035).<br>The following HRP-conjugated secondaries were used from Jackson Immunolabs Donkey anti-mouse (#715-035-150), Donkey anti-Rabbit (711-035-152), Donkey anti-Guinea Pig (#706-035-148).
Validation	All commercial antibodies were validated by the manufacturer as described in the manufacturer's handbook, by using positive controls to affirm reactivity. The Guinea Pig anti-ATF4 and -eIF2D antibodies made in our lab were validated using ATF4 and eIF2D mutants respectively in immunostaining and western blottiong experiments, by confirming that antibody reactivity was lost in null mutants.

Eukaryotic cell lines

Policy information about cell lines

Cell line source(s)	eIF2D mutant and control HAP1 cells were purchased from Horizon Discovery.
Authentication	HAP1 cells were validated by the manufacturer and by us using PCR to confirm deletion of eIF2D.
Mycoplasma contamination	Cells were not tested for mycoplasma.
Commonly misidentified lines (See ICLAC register)	No commonly misidentified cell lines were used in this study.

Animals and other organisms

Policy information about studies involving animals; ARRIVE guidelines recommended for reporting animal research

Laboratory animals	Supplementary Table 1 in the manuscript contains a detailed description of the genetic background and source of all Drosophila strains used in the study. Since DENR is on the X-chromosome, all Drosophila experiments involving DENR utilized only male animals whereas other experiments utilized a mix of males and females.<br>All animals of all genotypes in all experiments were of the same developmental stage. Animals in all experiments except Fig. 4, 5, 6 and Supplementary figure 5 were wandering 3rd instar. Animals in Fig. 4 and Supplementary Fig. 5 were monitored over development, with depicted images of pupa or pharate adults. Larvae in 5a, b were second instar, adult animals in 5c, d were monitored from day 0 - 25, and larvae in Fig. 6 were early 3rd instar.
Wild animals	No wild animals were utilized in this study.
Field-collected samples	No field-collected samples were utilized in this study.
Ethics oversight	Invertebrate organisms utilized in this study (Drosophila) do not require ethics oversight.

Note that full information on the approval of the study protocol must also be provided in the manuscript.

Magnetic resonance imaging

Experimental design

Design type	n/a
Design specifications	n/a
Behavioral performance measures	n/a

Acquisition

Imaging type(s)	n/a
Field strength	n/a
Sequence & imaging parameters	n/a
Area of acquisition	*State whether a whole brain scan was used OR define the area of acquisition, describing how the region was determined.*

Diffusion MRI ☐ Used ☒ Not used

Preprocessing

Preprocessing software	n/a
Normalization	n/a
Normalization template	n/a
Noise and artifact removal	n/a
Volume censoring	n/a

Statistical modeling & inference

Model type and settings	n/a
Effect(s) tested	n/a

Specify type of analysis: ☐ Whole brain ☐ ROI-based ☐ Both

Statistic type for inference (See Eklund et al. 2016)	n/a
Correction	n/a

Models & analysis

n/a	Involved in the study
☒	☐ Functional and/or effective connectivity
☒	☐ Graph analysis
☒	☐ Multivariate modeling or predictive analysis
